# Rational Design of Cellulosic Triboelectric Materials for Self-Powered Wearable Electronics

**DOI:** 10.1007/s40820-023-01094-6

**Published:** 2023-05-11

**Authors:** Xiangjiang Meng, Chenchen Cai, Bin Luo, Tao Liu, Yuzheng Shao, Shuangfei Wang, Shuangxi Nie

**Affiliations:** School of Light Industry and Food Engineering, Guangxi University, Nanning, 530004 People’s Republic of China

**Keywords:** Cellulose, Triboelectric nanogenerator, Triboelectric materials, Self-powered sensing, Wearable electronics

## Abstract

With the rapid development of the Internet of Things and flexible electronic technologies, there is a growing demand for wireless, sustainable, multifunctional, and independently operating self-powered wearable devices. Nevertheless, structural flexibility, long operating time, and wearing comfort have become key requirements for the widespread adoption of wearable electronics. Triboelectric nanogenerators as a distributed energy harvesting technology have great potential for application development in wearable sensing. Compared with rigid electronics, cellulosic self-powered wearable electronics have significant advantages in terms of flexibility, breathability, and functionality. In this paper, the research progress of advanced cellulosic triboelectric materials for self-powered wearable electronics is reviewed. The interfacial characteristics of cellulose are introduced from the top-down, bottom-up, and interfacial characteristics of the composite material preparation process. Meanwhile, the modulation strategies of triboelectric properties of cellulosic triboelectric materials are presented. Furthermore, the design strategies of triboelectric materials such as surface functionalization, interfacial structure design, and vacuum-assisted self-assembly are systematically discussed. In particular, cellulosic self-powered wearable electronics in the fields of human energy harvesting, tactile sensing, health monitoring, human–machine interaction, and intelligent fire warning are outlined in detail. Finally, the current challenges and future development directions of cellulosic triboelectric materials for self-powered wearable electronics are discussed.

## Highlights

- This review systematically discusses the interfacial properties of cellulosic material preparation processes, top-down, bottom-up, and composite processes.
- The rational design strategies of cellulosic triboelectric materials are summarized in detail, and the effects of different design strategies on the surface charge characteristics and charge density of cellulosic triboelectric materials are discussed.
- A comprehensive review of the research progress of cellulosic triboelectric nanogenerators in the field of self-powered wearable electronics.

## Graphical abstract

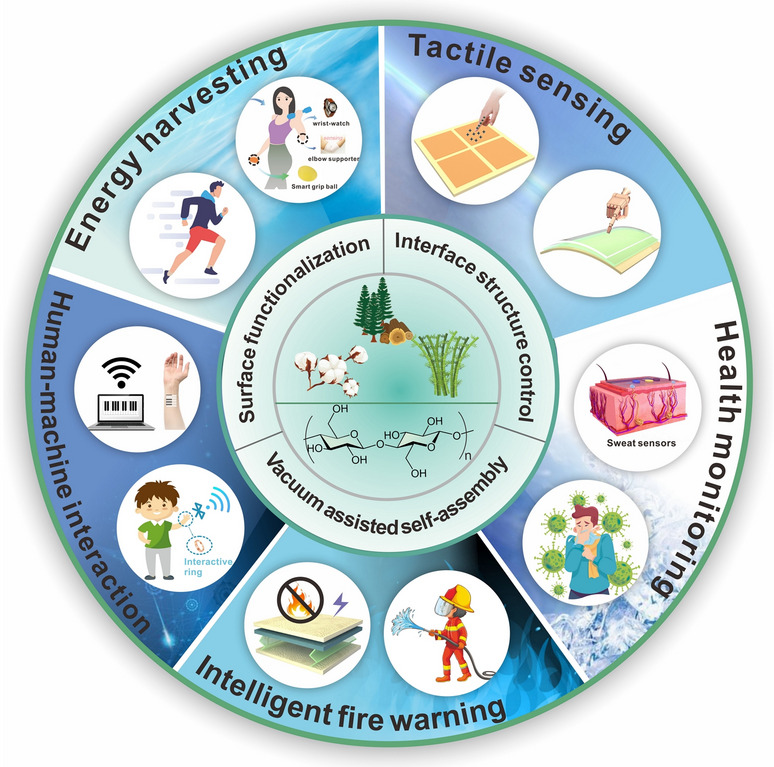

## Introduction

With the advent of the era of artificial intelligence, the miniaturization and portability of electronic products have made the industry of wearable smart products a high-profile industry, which makes the field of wearable electronics achieve considerable development in the past [1–3]. Wearable electronics have the capacity to process information in a non-invasive manner and are used in various detection applications [4]. In particular, wearable electronic products have brought in the interaction between humans and machines, and are an important part of the Internet of Things. They have a wide range of applications in the medical industry [5], sports industry [6], agriculture [7], and positioning [8]. Nevertheless, many wearable smart electronic devices with wireless systems use batteries as power sources [9]. Electronic devices’ miniaturization and functional diversification of has increased energy consumption, leading to issues such as short run times and frequent charging [10]. The emergence of triboelectric nanogenerators as a means of self-powered energy harvesting has breathed new life into wearable electronics [11]. The self-powered technology based on triboelectricity has the advantages of diverse material selection [12, 13], simple manufacturing process [14, 15], low cost [16], signal spontaneous [17], and a large application space in the collection of environmental energy and biological energy [18]. It offers new possibilities for the development of wearable electronic devices with extremely low power consumption and high flexibility [19]. It inevitably promotes the rapid development and wide application of next-generation wearable electronic devices and multifunctional artificial intelligence systems [20]. However, structural flexibility, long operating time, and wearing comfort have become key requirements for the widespread use of wearable electronics [21, 22]. Therefore, rational design and operational stability of sensing materials are crucial for the fabrication of wearable electronics.

Unlike common polymers, cellulose has excellent biocompatibility [23], flexibility [24], excellent mechanical properties [25], dielectric properties [26] and piezoelectric properties [27], and is a petroleum-based alternative product with degradable properties. The linear-chain structure of cellulose is composed of cyclic glucose molecules linked via covalent bonds, and its surface is rich in hydroxyl (-OH) reactive groups, resulting in a strong hydrogen-bonded network with modifiability [28]. The modifiability of cellulose segments makes it easy to be made into films [29], hydrogels [30], aerogels [31, 32], fabrics [33], and other materials for wearable electronics. Compared with rigid electronics, cellulosic wearable electronics have significant advantages in terms of flexibility, comfort, and functionality [34, 35]. In addition, unlike polytetrafluoroethylene (PTFE) [36], polyvinylidene fluoride (PVDF) [37] and polydimethylsiloxane (PDMS) [38] and other fluoroplastics commonly used today, cellulosic materials are better suited to meet the needs of environmental friendliness and sustainability [39]. The unique benefits of cellulose make it show good application potential in energy storage [40], energy harvesting [41, 42], water collection [43], human–machine interface unit [44], and flexible electronics [45].

Cellulosic triboelectric nanogenerators (TENGs) have recently undergone extensive development in the field of harvesting distributed energy. There have been several reviews covering the related topic of cellulosic TENGs. Zhang et al. [42], introduced the application and advantages of lignocellulosic fibers in TENG by analyzing the structural properties of lignocellulosic fibers. Zhao et al. [46], summarized the research methods for improving the power generation performance of cellulosic TENGs from the perspective of cellulosic materials and structure optimization of TENGs. With the in-depth study of cellulosic triboelectric materials, the processing and integration of cellulose piezoelectric and triboelectric nanogenerators for energy harvesting and other applications are systematically summarized [27]. Recent advances in cellulose paper-based triboelectric nanogenerators are also reported in detail [47]. It can be seen that cellulosic TENGs have great application potential. Self-powered wearable electronics prepared from cellulosic TENG can be well integrated into clothing, providing excellent breathability and good stretchability, which greatly improves the wearing comfort of users [48]. In addition, biodegradability [27] and flexibility [49] are important features of cellulosic self-powered wearable electronics. Moreover, the unique properties and good processability of cellulose make it suitable for large-scale fabrication of cellulosic functional materials for flexible electronics [50]. Reasonable design strategies are of great significance for expanding the application range of wearable electronics and can endow wearable electronic products with additional application characteristics, such as antifreeze [51], fire resistance [52], high sensitivity [53], electromagnetic shielding [54], high output performance [55]. At present, the preparation of cellulosic wearable sensing materials has derived a variety of design strategies. The bilayer hydrogel prepared by structural design shows good environmental stability, skin adaptability for wearable sensors, and bioelectrodes [56]. Nanoparticles can be added to the surface of cellulosic materials through physical doping strategies, which can effectively improve the sensitivity output of self-powered wearable electronics [57]. The strategy of surface modification can effectively endow cellulosic materials with other properties, such as acid, alkali resistance, and durability [58]. These preparation strategies have laid a research foundation for the development of wearable electronics applied in various fields.

**Fig. 1:**
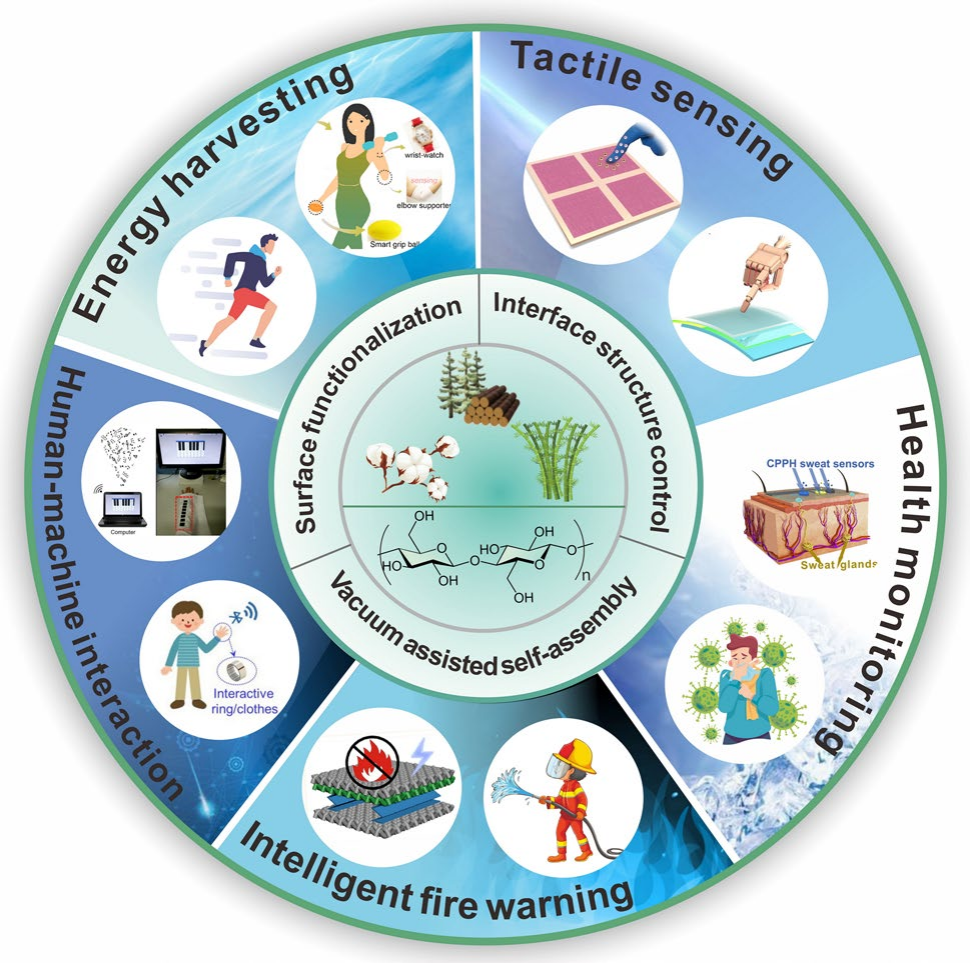
Cellulosic self-powered wearable electronics. Reproduced with permission from Ref. [59], Copyright 2020, American Chemical Society; Ref. [60], Copyright 2022, Elsevier; Ref. [61], Copyright 2017, American Chemical Society; Ref. [62], Copyright 2022, Wiley–VCH; Ref. [63], Copyright 2020, American Chemical Society; Ref. [64], Copyright 2020, Elsevier; Ref. [23], Copyright 2022, Elsevier

Herein, this paper aims to provide new perspectives for the preparation of self-powered wearable electronics, and the research progress of cellulosic wearable electronics is reviewed (Fig. 1). The characteristics of the material preparation process, the preparation strategy, and the influence of different preparation strategies on the triboelectric properties are comprehensively introduced. Among them, the effects of different preparation strategies on triboelectric positive polarity, triboelectric negative polarity, and charge density of cellulosic triboelectric materials are discussed, which can help to prepare wearable electronic products with stable triboelectric signal output. In particular, the latest application development of cellulosic wearable electronics is reviewed, such as research in the fields of human energy harvesting, tactile sensing, health monitoring, human–machine interaction, and intelligent fire warning. Finally, the current challenges and future developments of cellulosic wearable electronics are summarized and discussed.

## Interfacial Characteristics of Cellulosic Triboelectric Materials

**Fig. 2:**
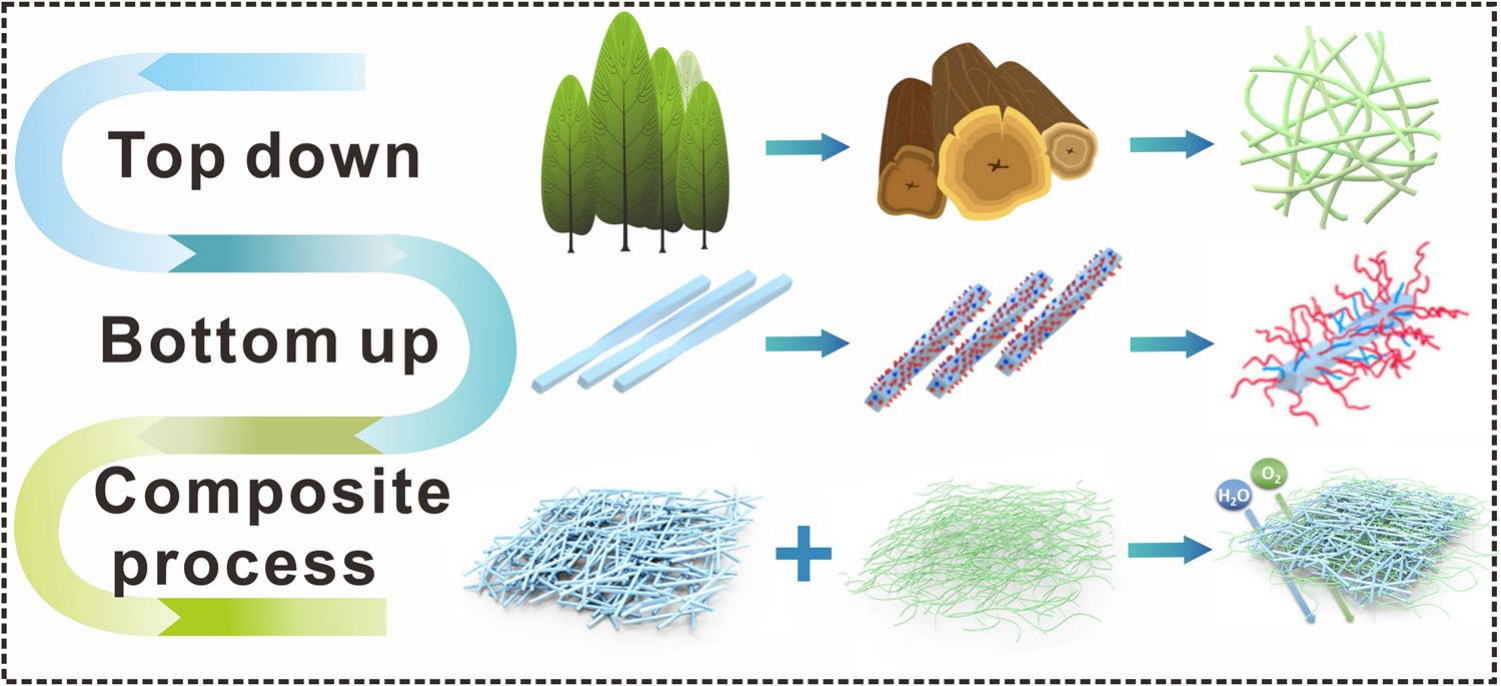
Interface properties during the construction of cellulosic materials. Reproduced with permission from Ref. [80], Copyright 2018, American Chemical Society; Ref. [29], Copyright 2019, American Chemical Society

Recently, a wide variety of cellulosic materials have been used as triboelectric materials, including aerogels [65], hydrogels [66], thin films [67], and structural materials [68]. During the processing of nanocellulose advanced materials, the original surface chemistry and interfacial interactions can change significantly [69]. The interfacial properties of research materials play an important role in the preparation of triboelectric materials, affecting the characteristics of triboelectric materials such as polarization [70], breakdown strength [71], and defects of composite materials [72]. Many people have taken the perspective of interface design to increase the interface area [73] and improve the dielectric constant [74] of the composite material, thus improving the triboelectric properties of the material, which has the effect of enhancing the charge density [75] or improving the charge characteristics [76]. The investigation of top-down, bottom-up, and composite interfacial properties is important for the enhancement of triboelectric properties in certain functional materials (structural materials, thin films, filaments, aerogels, and foams) for better performance in the field of wearable electronics [77–79]. This section introduces the top-down, bottom-up, and composite interface properties of cellulosic materials (Fig. 2), and provides design ideas for cellulosic triboelectric materials for self-powered wearable electronics from different prepared interfaces of cellulosic materials.

### Interfacial Characteristics of Cellulose in Top-down Processes

Cellulose is a sustainable and renewable resource derived mainly from the natural environment, such as wood, bagasse, cotton wool, and algal organisms [81, 82]. Preparation of microcellulose from virgin materials is a top-down process at the macroscopic level. Mechanical, chemical, and enzymatic treatments are commonly used to extract nanocellulose from natural sources [83–86]. In the top-down preparation process, a simple chemical pretreatment employing acidified sodium chlorite solution and potassium hydroxide (KOH) solution is used to remove lignin and part of the hemicellulose from wood flour, followed by a mill-based nanofibrillation of the prepared cellulose pulp to produce cellulose nanofibers (CNFs) of uniform width [87]. CNFs are usually prepared by mechanical methods, mainly including homogenization, refining, grinding, ball milling, and high-intensity ultrasonic treatment, among which homogenization and grinding are the most commonly used methods for preparing CNFs [88]. For the preparation of CNC, acid hydrolysis or enzymatic hydrolysis is often used to prepare CNC [89]. Strong-acid hydrolysis hydrolyzes the amorphous regions of cellulose, resulting in short CNCs with low aspect ratios but high specific surface areas and relative crystallinities [90]. For the top-down process, the phenomenon of dispersoid aggregation is prone to occur. Grafting of negatively charged sulfate half-ester groups onto the surface of CNC particles by acid hydrolysis is used to prevent electrostatic repulsion between particles and thus aggregation in aqueous suspensions [91]. The promotion of nanofibrillation via surface chemical modification is another effective method for the top-down preparation of nanocellulose [92].

**Fig. 3:**
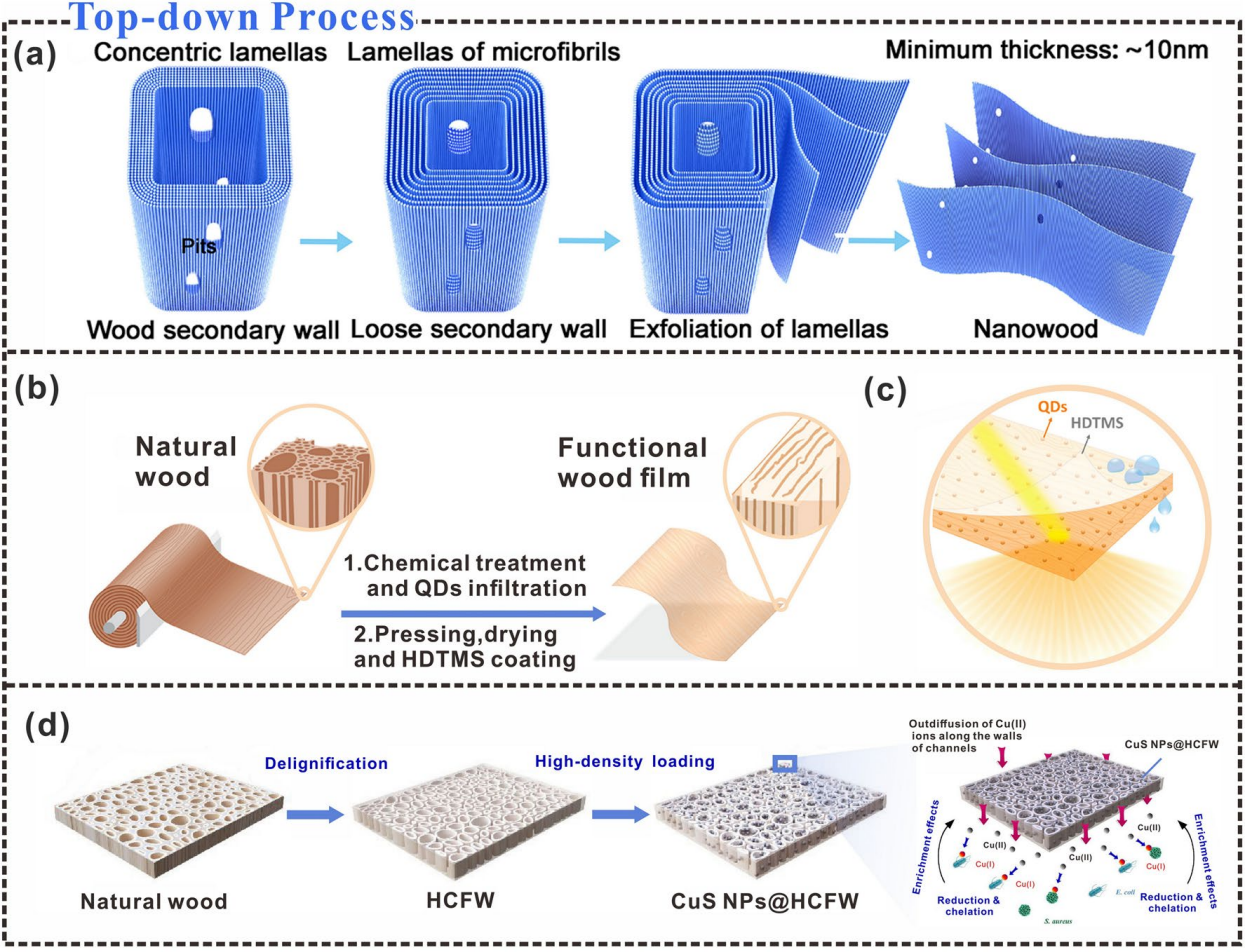
Interfacial characteristics of cellulose in top-down processes. **a** The top-down cell wall stripping process. Reproduced with permission from Ref. [94]. Copyright 2021, American Chemical Society. **b** Top-down construction of freestanding cellulose nanomembranes. **c** Cellulose nanofilms are used in optical lighting materials. Reproduced with permission from Ref. [97]. Copyright 2020, American Chemical Society. **d** Top-down diagram of CuS NPs@HCFW preparation. Reproduced with permission from Ref. [98]. Copyright 2020, Elsevier

Compared with mechanical nanofibrillation, the combination of TEMPO-mediated oxidation modification and mechanical treatment has more advantages. This is because TEMPO oxidation can introduce negative charges on the surface of CNFs, resulting in a change in charge transfer. Subsequent mechanical nanofibrillation requires less energy consumption, making nanofibrillation relatively easy [93]. During top-down material construction, the use of ultrasonic means to partially separate independent cellulose nanomembranes can effectively construct sub-nanochannels, which can facilitate ion transport (Fig. 3a) [94]. Furthermore, cellulose is rich in functional groups, and its surface charge density can be easily tuned via chemical modification. CNFs can be exposed by shedding lignin and hemicellulose during nanofibrillation. Surface charges on the CNF interface aligned to the fiber direction control the ion transport in an ideal ion separation, which facilitates the directional transport of ions [95]. Ion transport can also be controlled by adjusting the surface charge, which has excellent prospects in electronic skin and energy harvesting and storage systems [96].

Top-down material construction results in high mechanical compressibility and elastic recovery, because it breaks the thin cell wall of the natural plant and allows siloxane coating to grow on the scaffold surface [99]. Furthermore, owing to the breakdown of the thin cell walls of native plants and the removal of lignocellulose and half of the hemicellulose (Fig. 3b), the prepared cellulose wood films have the potential for optical applications (Fig. 3c) [97]. In addition, in the top-down production process of cellulosic materials, CNF can be better modified by interfacial hydrophobicity, which can help cellulosic triboelectric materials to obtain stable triboelectric output in a humid environment and better collect blue energy [78]. Removing lignin and hemicellulose from wood is another effective way to construct the porous structure of wood. The top-down preparation of cellulosic porous materials can increase the sensing area of the material to spoilage gases, thereby realizing the anti-corruption of food spoilage gases (NH_3_) high response sensing [100]. Achieving high anisotropy in current aerogels usually requires additional strategies. However, the dissolution and regeneration of nanofibers can increase cell-wall porosity and create highly anisotropic structures [101]. High-porosity nano-fibrillated networks exhibit high compressive strength. After the in-situ synthesis of metal (Ag) and metal oxide (TiO_2_) nanoparticles, conductive polymers are deposited on them and carbonized during the assembly process to produce conductive aerogels. This top-down material preparation process provides a new perspective for the development of anisotropic composites. The synthesis of an all-cellulose framework (HCFW) of anisotropic microchannels via a top-down construction pathway shows great potential for antibacterial activity, pH-responsive drug delivery, and antibiotic removal applications, owing to its capacity to guide the directional assembly of CuS nanoparticles (NPs) along the microchannel walls (Fig. 3d) [98]. In addition, in top-down material construction processes, carboxymethylation of cellulose followed by partial etherification of hydroxyl groups can lead to controllable surface charges and substituted chain segments, providing corresponding strategies for the development of commercial packaging materials, flexible wearable electronic devices, and biomaterials [102].

### Interfacial Characteristics of Cellulose in Bottom-up Processes

**Fig. 4:**
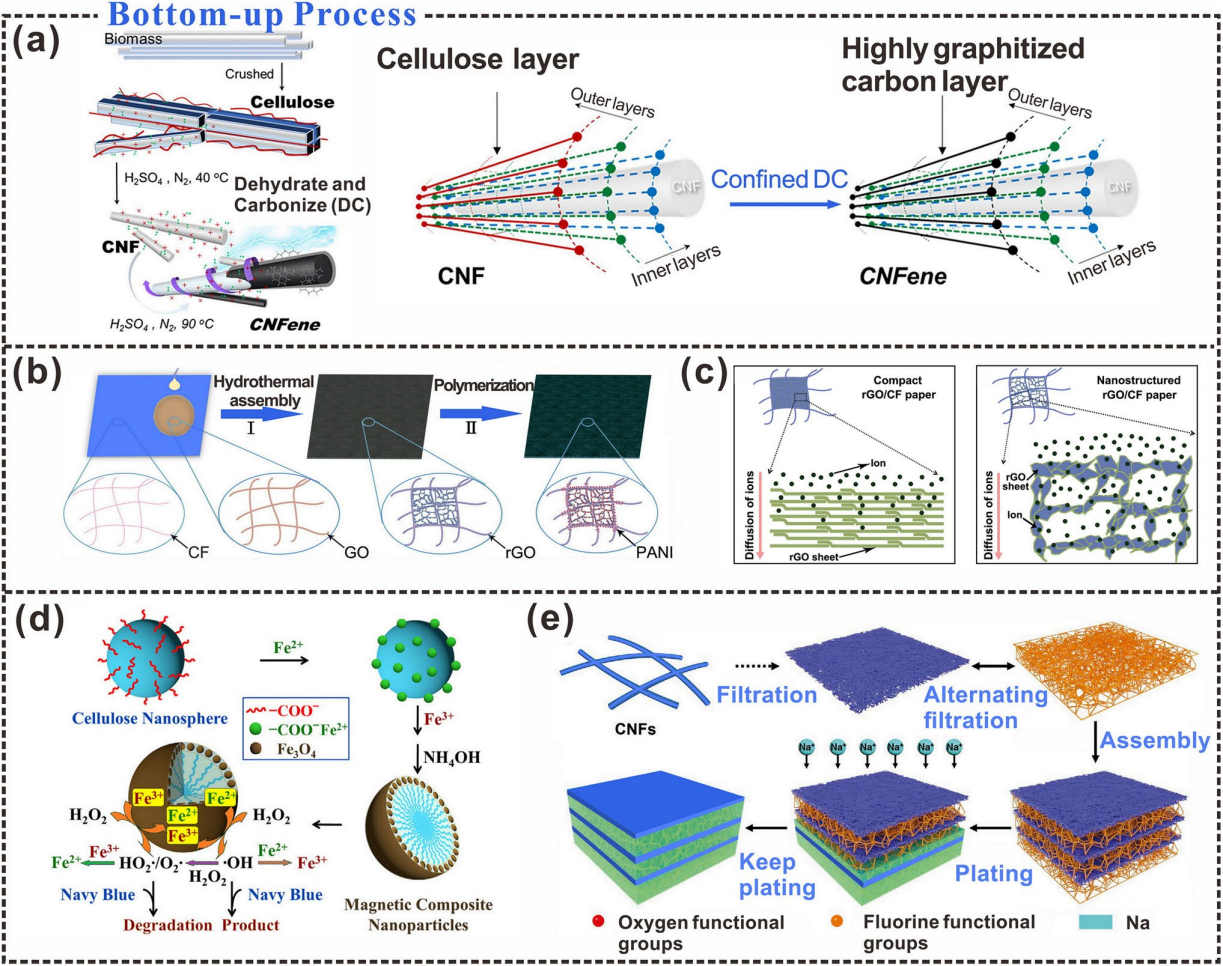
Interfacial characteristics of cellulose in bottom-up processes. **a** Schematic diagram of the extraction process of CNF and the mechanism of carbonization of the outer layer of CNF into highly graphitized carbon. Reproduced with permission from Ref. [107]. Copyright 2021, American Chemical Society. **b** Schematic diagram of pani-RGO/CF composite paper preparation. **c** Schematic illustration of ion diffusion in nanostructured rGO/CF composite paper. Reproduced with permission from Ref. [108]. Copyright 2014, Wiley–VCH. **d** Magnetic iron oxide was anchored on cellulose nanospheres by the co-precipitation method. Reproduced with permission from Ref. [109]. Copyright 2015, Elsevier. **e** The preparation process of conductive composite paper. Reproduced with permission from Ref. [110]. Copyright 2022, Wiley–VCH

Bottom-up material construction is also an interesting method of choice. Conceptually, it refers to the process of preparing cellulose from microfine fibers into cellulosic materials, involving the synthetic construction of cellulose chains, followed by oligomerization-induced self-assembly to form hierarchically organized materials with tunable properties [103–105]. Cellulose is the most abundant renewable natural polymer on Earth and has received considerable attention for its environmental compatibility and self-assembly properties [96, 106]. Although cellulose itself has many excellent properties, its lack of electrical conductivity limits its potential for use in energy storage, catalysis, and sensing. It has been reported that continuous reaction processes can be controlled by sequestering oxygen in a bottom-up process. Local molecular chains of the CNFs can be converted to highly graphitized carbon (Fig. 4a), resulting in the direct extraction of intrinsically conductive CNFs and graphene crystallites (collectively referred to as CNFenes) from biomass [107]. This addresses the issue of the electrical non-conductivity of nanocellulose and provides more possibilities for the application of cellulose in the energy field. Bottom-up processes of preparing cellulosic materials can not only influence their properties by controlling the reaction but also make full use of their porous structure. Cellulose paper contains voids. Graphene oxide (GO) can be assembled into reduced graphene oxide (rGO) in the pores of cellulose paper using hydrothermal processes to form an rGO/CF composite paper (Fig. 4b). The presence of the cellulose fiber (CF) network and porous structure in the paper (Fig. 4c) makes the transportation of electrons and ions easier and prevents the aggregation of rGO sheets in the paper pores, resulting in a cellulose substrate with excellent energy-storage properties [108]. The increased electrical conductivity of cellulosic materials allows cellulosic materials to be used as flexible electrode materials in triboelectric nanogenerators, solving the bottleneck of poor flexibility of other electrode materials [67].

Chemical co-precipitation is also a commonly used bottom-up preparation method. Iron oxide (Fe_3_O_4_) can be anchored on the surface of carboxy cellulose nanospheres via chemical co-precipitation to prepare magnetic composite nanoparticles (MNPs; Fig. 4d) [109]. MNPs can be efficiently recycled and reused using an external magnetic field and can effectively degrade chemical pollutants. Doping can also help construct a stable reaction interface. Alternate assembly of fluorine-super-doped carbon nanotubes with CNFs generates a stable reaction interface with a self-correction function. Thereby realizing a "bottom-up" sodium-deposition pattern and the successful preparation of a periodic conductive/dielectric composite paper (Fig. 4e) [110]. The rapid development of the internet of things (IoT) and flexible electronics necessitates flexible and efficient energy units. A bottom-up dual growth strategy, based on paper microfluidics and wax printing, has been used to overcome the vertical conductivity problem of the conductive/dielectric composite paper, resulting in not only self-charging functions but also self-powered electrical aggregation and sensing [111].

### Interfacial Characteristics of Cellulose Composites

Both the preparation of raw fibrillated cellulose and the customization of cellulose composites are inseparable from an aqueous environment. Cellulosic composites tend to reduce the swelling capacity, compliance, wet flexibility, and bonding potential of chemical pulp fibers after water removal or drying, a phenomenon known as keratinization [112]. The keratinization of nanocellulose has a significant impact on the performance and life cycle of cellulosic materials and causes fiber agglomeration, making it difficult to redisperse cellulose [113]. The aggregation and dispersion of cellulose can be determined via the interfacial charge properties [114]. Dispersibility is affected by surface-charge density. The dispersion of nanocellulose can be promoted by increasing the electrostatic repulsion or steric hindrance between the nanocelluloses by introducing surface charges or long-chain molecules [115]. The surface charge can induce fibril alignment and gel transition in an aqueous environment, thereby fixing the nanostructure of the material and resulting in uniformly dispersed filaments [116]. Preparation of cellulose composites using the interaction between water and the cellulose interface (aggregation and dispersion) is noteworthy, as it can produce not only films with good dispersibility and uniformity but also regenerated fiber films with ultra-high toughness and transparency, which inspires the application of cellulose functional materials in the field of wearable sensors [117].

**Fig. 5:**
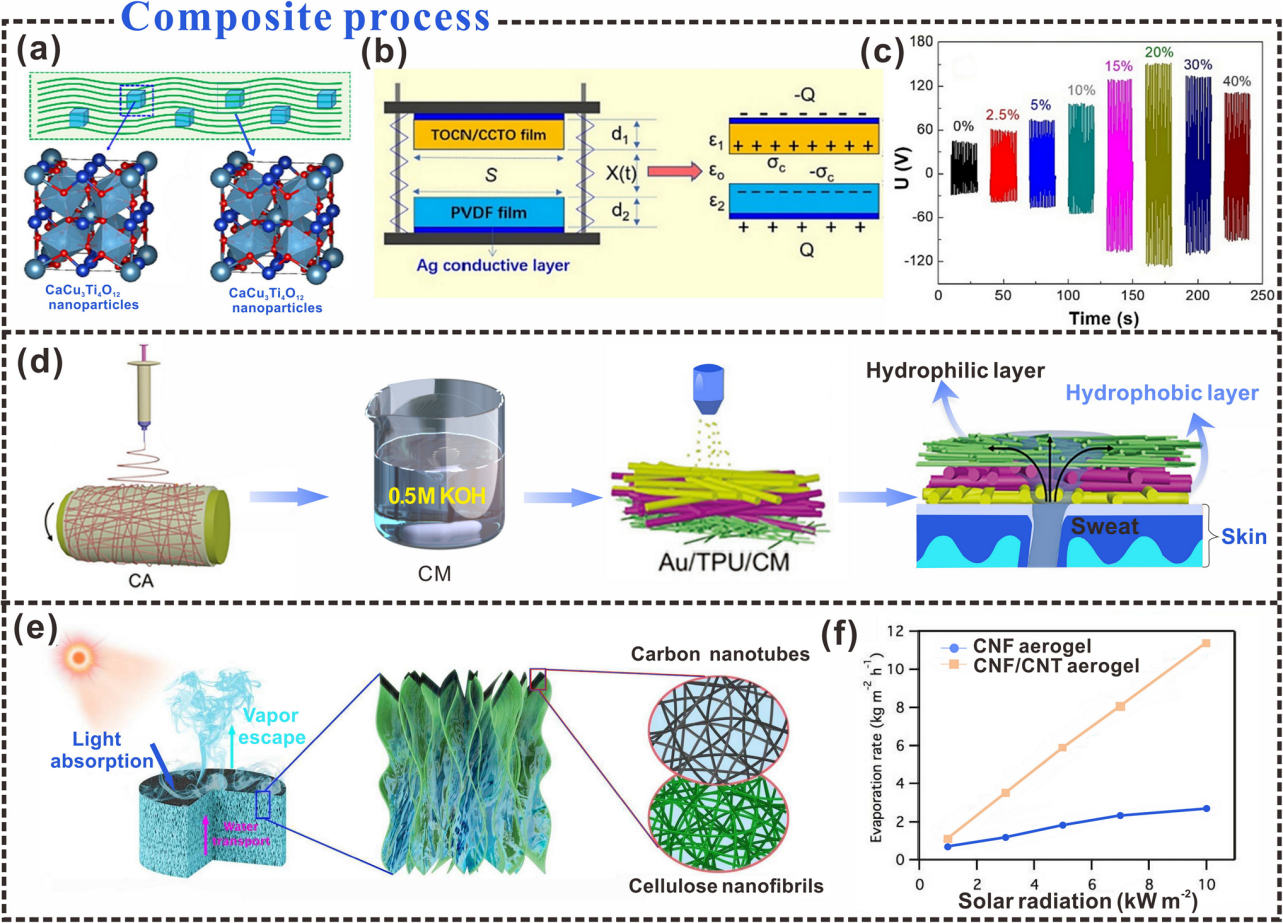
Interface properties of cellulosic composites. **a** Schematic diagram of calcium copper titanate added to TEMPO-oxidized cellulose nanofibers. **b** TENG is composed of TOCN/CCTO films. **c** Triboelectric output measured at different CCTO contents. Reproduced with permission from Ref. [65]. Copyright 2022, Elsevier. **d** Flow chart of the fabrication of the sweat-sensing electronic skin. Reproduced with permission from Ref. [121]. Copyright 2022, Wiley–VCH. **e** All-nanofiber CNF-CNT aerogel for solar steam generation. **f** The density and porosity of the CNF and CNF-CNT bilayer aerogels are both extremely low density below 0.01 g cm^−3^ and high porosity above 99%. Reproduced with permission from Ref. [122]. Copyright 2017, American Chemical Society

The aggregation of nanoparticles during the preparation of common triboelectric materials hinders their improvement. Avoiding material aggregation and improving dispersion can effectively increase the triboelectric properties of the material [118, 119]. For the preparation of cellulosic triboelectric materials, the quality of dispersion during material preparation is also one of the factors affecting the triboelectric output. Song et al. [65], demonstrated the effect of agglomeration of copper calcium titanate (CCTO) nanoparticles on the triboelectric output of cellulosic triboelectric materials by discussing the dispersion of CCTO nanoparticles in TEMPO-oxidized cellulose nanofibers (TOCN) (Fig. 5a). The TENG device was constructed with the TOCN/CCTO film as the triboelectric positive layer and the PVDF film as the triboelectric negative layer (Fig. 5b). CCTO films were prepared by changing the content of CCTO nanoparticles. It was found that the suspension of CCTO and TOCN was well dispersed when the content of CCTO reached 20%. The contact electrification is carried out with a CCTO film with a content of 20%, and the voltage can reach up to 152 V (Fig. 5c). In addition, a mixture of water and tert-butanol (TBA) was used to prepare a new TOCN aerogel to prevent the extrusion agglomeration of cellulose nanofibril due to volume expansion of water dispersion during the freezing process [120]. With the further addition of TBA, the surface tension of the suspension was further reduced. The reduction of surface tension of TOCN dispersion is beneficial to reduce the aggregation of cellulose nanofibrils during freezing, which leads to aerogels with higher specific surface area and excellent triboelectric properties.

From the perspective of material dispersion, a variety of ideas have been provided for material preparation, and the aggregation phenomenon of materials will also be required on some specific occasions. Under aqueous conditions, oppositely charged nanocellulose was fabricated into continuous fibers by interfacial nanoparticle complexation (INC). The fabrication of other oppositely charged nanoparticle pairs into continuous fibers could also be explored [123].

An in-depth study of the characteristics of cellulose composites can expand the application potential of cellulosic materials. Exploring the transport mechanism between water and cellulose during composite preparation could extend the application of cellulosic materials to the field of sensors, such as gold/thermoplastic polyurethane/cellulose film (Au/TPU/CM) electrodes with double porosity and surface-energy gradients for the production of electronic skin for sweat sensing (Fig. 5d) [121]. Unidirectional water transport from the skin to the sensor surface can be achieved through the synergy of the porosity and surface-energy gradients of the Au/TPU/CM film structure (Fig. 5e). In addition, aerogels composed of nanofibers (CNFs and carbon nanotubes (CNTs)) with bilayer structures can be designed for compressible and efficient solar steam generators (Fig. 5f) [122]. The aerogel based on the double-layer structure provides excellent porosity, which promotes the speed of water vapor passage and improves the efficiency of steam power generation. Such high-performing double-layered solar steam power-generation devices can provide a new design strategy for energy development and utilization.

## Properties Modulation of Cellulosic Triboelectric Materials

**Fig. 6:**
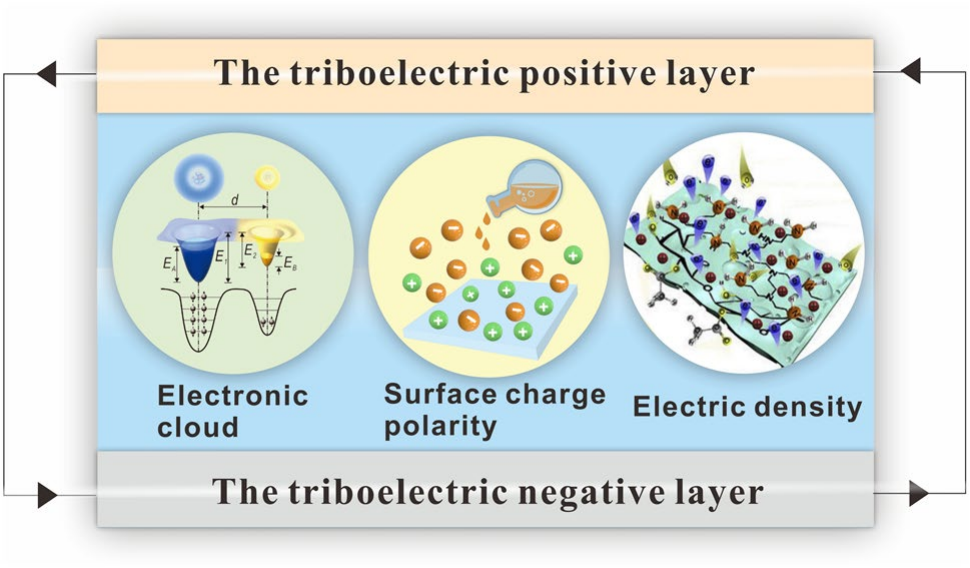
Properties modulation of cellulosic triboelectric materials. Reproduced with permission from Ref. [124]. Copyright 2018, Wiley–VCH. Reproduced with permission from Ref. [126]. Copyright 2020, Elsevier

As mentioned above, from the perspective of interface properties, discussing the impact of interface enhancement on material preparation will help provide ideas for the fabrication of cellulosic wearable electronics. However, the performance modulation of cellulosic triboelectric materials has an important impact on the stability and high performance of triboelectric output. In general, polymer–polymer contact electrification (CE) is explained by electron clouds [124, 125]. Starting from the influence of charge transfer in the process of contact electrification, the performance modulation of cellulosic triboelectric materials is discussed, which is conducive to the preparation of cellulosic self-powered wearable electronics with stable output. This section mainly discusses the effects of performance modulation of cellulosic triboelectric materials on the triboelectric positive polarity, triboelectric negative polarity, and charge density of cellulosic materials (Fig. 6).

### Triboelectric Positive Polarity

**Fig. 7:**
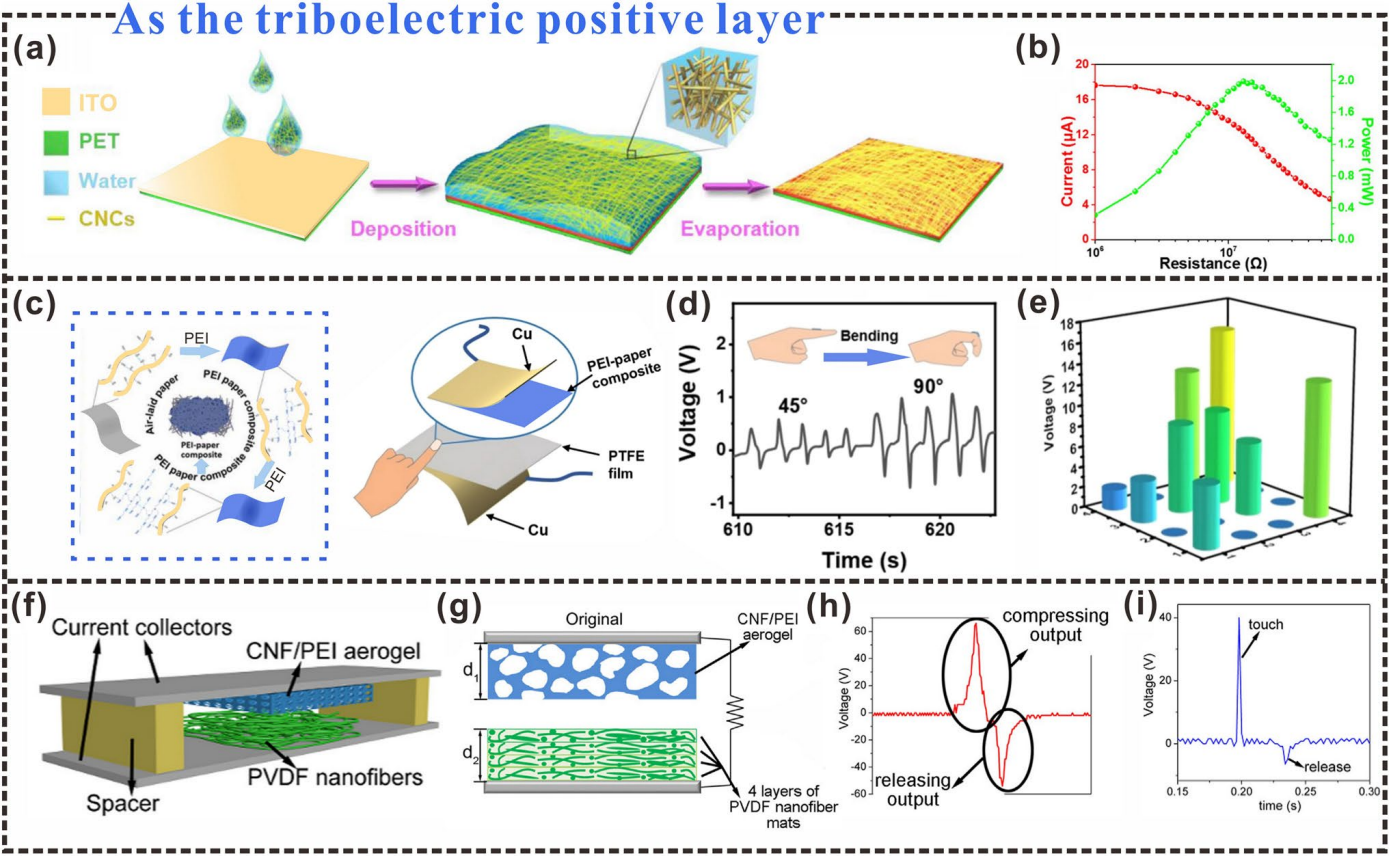
Cellulosic triboelectric materials as positive triboelectric layers. **a** Schematic diagram of CNC/ITO electrode. **b** Dependence of output current and corresponding power of TENG on external load resistance at 20 m s^−1^ wind speed. Reproduced with permission from Ref. [132]. Copyright 2018, Elsevier. **c** Schematic diagram of the fabrication of PEI-paper composites. **d** Detects the voltage response to finger bending. **e** Voltage signal distribution under an applied force. Reproduced with permission from Ref. [133]. Copyright 2022, Elsevier. **f** Schematic illustration of a TENG fabricated by pairing a CNF/PEI airgel film with a PVDF nanofibrous mat. **g** Schematic diagram of the working principle of the CNF/PEI-PVDF TENG. **h** Typical output triboelectric voltage signal during one compression and release cycle. **i** Triboelectric signals can be easily generated by finger taps. Reproduced with permission from Ref. [134]. Copyright 2018, Elsevier

According to the triboelectric material sequence, unprocessed cellulosic materials tend to lose electrons during contact with other materials, thus exhibiting triboelectric positive polarity [127]. Therefore, enhancing the triboelectricity of cellulose is beneficial to improve the performance of cellulosic triboelectric materials in practical applications. It has been reported that by using regenerated cellulosic materials as the triboelectric layer, the TENG prepared has high triboelectric properties, and the power density can reach 300 W m^−2^, which has good application potential [128]. At present, cellulosic materials used as positive triboelectric layers mainly include paper [129], film [130], aerogel [79], hydrogel [131]. The use of cellulose suspension to make triboelectric layers is one of the common methods. CNC/ITO films with good transparency can be prepared by uniformly coating CNC suspension on the surface of indium tin oxide (ITO) films. The prepared CNC/ITO films can be used as triboelectric positive layers, ad fluorinated ethylene propylene (FEP) films as the triboelectric negative layers (Fig. 7a) [132]. After several up-and-down vibration cycles, positive triboelectric charges accumulated on the surface of the CNC, at which point the amount of charge on the FEP film and the CNC reached a balance, thus realizing the activation state of the TENG with stable voltage output (Fig. 7b).

Yao et al. developed a novel method for TENG fabrication from cellulose suspension [135]. They oxidized and mechanically homogenized wood pulp to obtain cellulose nanofilaments, which were uniformly dispersed in water to prepare CNF hydrogels. The CNF hydrogels were filtered and dried under pressure to obtain transparent and flexible CNF films. A CNF-based TENG was then fabricated by assembling the CNF and FEP films, and the charge-transfer efficiency of the power board was as high as 98%, indicating a very high internal charge-output efficiency. There have been many similar efforts to enhance triboelectric positive polarity generation and promote charge transport in cellulosic papers. Polyethyleneimine (PEI) is a highly branched polymer containing approximately 25% primary, 50% secondary, and 25% tertiary amine groups and can provide more amine groups than conventional amino compounds (Fig. 7c), which can be used to improve the triboelectricity of cellulose fiber paper. PEI–paper composites can be prepared using cellulose paper as the backbone and PEI as the liquid filler, thus enhancing the triboelectric polarity of the cellulosic paper [133]. The triboelectric energy generated during the material contact and separation process is used to detect the voltage response caused by finger bending (Fig. 7d). Under the action of force, the voltage distribution signal has also been fully displayed and has been successfully used in pressure sensors (Fig. 7e).

Self-assembly of a layer of polydopamine on the surface of chewing gum wrapping paper is another way to prepare a cellulosic triboelectric layer [136]. Polyvinylidene fluoride (PVDF) film was used as a triboelectric negative layer. The fabricated arched-structure TENG can be used in anti-corrosion and anti-fouling coatings and self-powered machines in the ocean, besides wind-power harvesting. The in-situ self-polymerized chewing-gum wrapper is more likely to lose electrons when rubbed on the PVDF triboelectric layer, exhibiting a high triboelectric output, with short-circuit current and output voltage being increased by more than 3.5 times, and the surface polarity of the triboelectric layer was successfully improved.

Unlike the normal contact-separation mode, the sliding-friction TENG surface causes periodic contact separation, resulting in the lateral separation of charge centers, which drives the voltage drop of the electron flow, when, for example, cellulose paper and Teflon are used as positive and negative tribological layers, respectively. Xia and his collaborators first proposed a sliding paper TENG (SP-TENG) that was built based on an in-plane charge disintegration mechanism, which utilizes related sliding between two interacting facades [137]. The carbon ink is applied to the paper surface in the form of a surface coating, forming a paper-carbon-paper structure, with the paper acting as a triboelectric positive layer and the carbon ink acting as a conductive electrode, aiding the stacked SP-TENG in obtaining a better triboelectric output. The SP-TENG achieves a larger charge with a peak instant power of approximately 18.4 W m^−2^. SP-TENG has strong flexibility and portability and has been successfully used in self-powered speed sensing and force sensing.

With the development of cellulosic triboelectric materials, multidimensional cellulosic triboelectric materials have gained increasing attention as triboelectric positive layers. Three-dimensional materials, such as aerogels, have been studied as triboelectric positive layers. Aerogels cross-linked between PEI and CNF, prepared via freeze-drying, have been used as triboelectric positive layers (Fig. 7f) [134]. The strongly electron-donating amino groups were introduced during a reaction between CNF and branched PEI, which enhanced the electrostatic induction and triboelectricity of the triboelectric layer. The TENG made of CNF/PEI aerogel and four-layer PVDF nanofiber mat exhibited 18.3 and 97.6 times higher output voltage and power density, respectively. The triboelectric output has been greatly improved. During the contact separation process, an obvious triboelectric output signal can be observed through a compression and release cycle (Fig. 7g, h). The CNF/PEI aerogel TENG can not only effectively detect the motion state of arm bending or stepping, but also monitor the motion state of light force such as finger tapping, which has high sensitivity (Fig. 7i). Cellulosic aerogel structures can also be prepared via 3D printing. Triboelectric micro-3D patterns and nanoporous cellulose aerogels can be fabricated via printing [138]. To further improve triboelectricity and roughness, CNF-based 3D micro/nano-layered pattern structures were fabricated by freeze-drying process and post-annealing process. The periodic contact of the triboelectric positive and negative layers results in an alternating current output signal in the external circuit. The harvested energy can drive 88 LED lights to be used as self-powered motion sensors to detect finger and foot movements. It can be seen that by enhancing the triboelectric positive polarity of the cellulosic triboelectric material, the charge transfer can be effectively promoted, thereby improving the triboelectric output.

### Triboelectric Negative Polarity

**Fig. 8:**
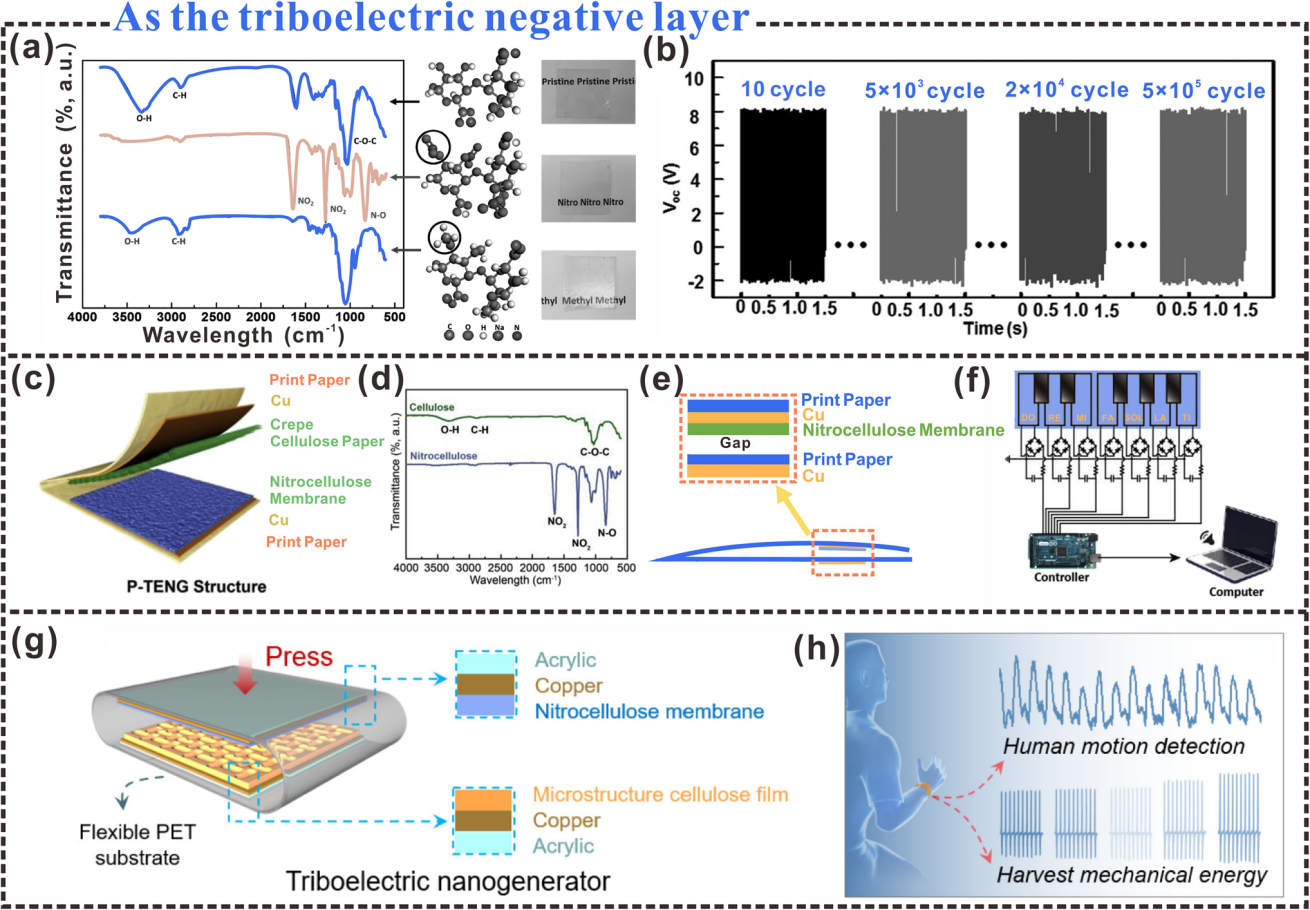
Cellulosic triboelectric materials as negative triboelectric layers. **a** FTIR spectra and molecular structures of raw CNF (top row), nitro-CNF (middle row), and methyl-CNF (bottom row). **b** The long-term voltage output of TENGs is made from nitro-CNF/methyl-CNF pairs. Reproduced with permission from Ref. [142]. Copyright 2017, Wiley–VCH. **c** Triboelectric nanogenerators based on CCP and NCM. **d** FTIR spectra of CCP and NCM. **e** P-TENG composition. **f** Interactive application of P-TENG and computer to realize paper piano performance. Reproduced with permission from Ref. [143]. Copyright 2019, Elsevier. **g** Structure of an M-CF-based triboelectric nanogenerator. **h** Potential applications in motion detection and energy harvesting. Reproduced with permission from Ref. [153]. Copyright 2019, American Chemical Society

By introducing different functional groups to tune the triboelectric properties of cellulose, it is possible to fabricate potential TENGs of all cellulosic triboelectric materials. Cellulose is slightly positively charged in the triboelectric sequence [12, 139]. It is precise because of the weak polarization tendency of natural cellulose that results in the limited ability to generate charges on the cellulose surface. However, since cellulose is rich in hydroxyl groups, chemically introducing new functional groups to adjust the triboelectric polarity of cellulose is an effective way to expand green energy harvesting devices [140]. Table 1 lists the performance comparison of cellulosic materials as triboelectric negative layers and other polymers as triboelectric negative layers. Nitrocellulose thin films are often used as triboelectric negative layers, providing a viable strategy for commercial cellulosic materials for real-time sensing applications [141]. Yao et al. [142], chemically functionalized the decomposed cellulose to prepare cellulosic triboelectric negative layer (Fig. 8a). Nitrified-CNF was prepared by treating CNF fibers with a nitric acid mixture composed of HNO_3_, H_2_SO_4_, and water. Due to the addition of nitro (-NO_3_) to improve the electron affinity of cellulose molecules, the prepared Nitro-CNF has a charge density of 85.8 µC m^−2^. The charge density values were corresponding to 71% of that for FEP, the most triboelectric negative material, while pristine CNF could only reach 11% of FEP the triboelectric output of the CNF films after nitrification was significantly enhanced (Fig. 8b), and the triboelectric surface potential increased by a factor of 4–6. Chen et al. [143], constructed a TENG using a nitrocellulose membrane (NCM) and crepe cellulose paper (CCP) as the negative and positive triboelectric layers, respectively (Fig. 8c). Upon contact between the positive and negative tribological layers, the CCP tends to lose electrons and develop a positive surface charge. The NCM surface, which contains electron-withdrawing nitrate groups (Fig. 8d), tends to gain electrons and develop a negative charge. A paper-based triboelectric nanogenerator was prepared using NCMs and CCP (Fig. 8e), which can be used for human–computer interaction applications with a computer for paper piano playing through further connections that have high application prospects (Fig. 8f).

**Table 1:** The performance comparison of cellulosic materials as triboelectric negative layers and other polymers as triboelectric negative layers

Triboelectric negative layer	Materials property					
	Biodegradability	Sensing performance	Lit LED lights	Recycling characteristics	Application	References
Fluorinated-cellulose	Yes	Yes	20	2000 times (the voltage can remain stable after 15 days)	Human motion sensing	[144]
Cellulose nanocrystalline flakes (CNCF) Composites	Yes	None	100	–	Energy harvesting	[145]
TiCl_4_-AZO-CNF	Yes	None	–	648,000 times	Mechanical energy harvesting	[146]
Nitrocellulose paper	Yes	Yes	–	12,000 times	Self-powered wireless transmission systems	[141]
Cellulose/PDMS film	Yes	Yes	12	3600 times	Human Motion Sensing	[147]
Polyimide (PI)	None	Yes	–	–	Self-powered sensor	[148]
PDMS-PTFE composite membrane	None	Yes	150	10,000 times	Human body temperature thermometer	[149]
Polytetrafluoroethylene (PTFE)	None	None	5	–	Energy harvesting	[150]
Polydimethylsiloxane (PDMS)	None	None	60	–	Energy harvesting	[151]
Polyvinylidene fluoride (PVDF)	None	None	80	14,400 times	Energy harvesting	[152]

Cellulosic materials with electron-withdrawing groups are gradually gaining prominence in energy-harvesting systems. The use of cellulose as a negative triboelectric layer is helpful for the preparation of full-paper TENG, improving the application potential of flexible and miniaturized green electronics [154]. Cellulose paper/polypyridine composite was used as the positive triboelectric layer, and nitrocellulocellulosic film (NCM) was used as the negative triboelectric layer for the manufacture of P-TENG. When the positive and negative triboelectric layers of the arch P-TENG contact each other, electrostatic induction will be generated on the surface of the triboelectric layer. At this time, negative charges will be generated on the nitrocellulose membrane NCM, and the electric energy generated by the contact between the nitrocellulose membrane NCM and the triboelectric positive layer successfully drives 30 LED lamps, which has a good energy harvesting effect. In addition, P-TENG can also be combined with supercapacitors to form a self-powered power system, successfully integrating energy harvesting and electrical energy storage into a miniaturized electronic product with both practicality and flexibility.

There are also many examples of cellulosic materials as triboelectric negative layers in the field of human motion detection and healthcare. Cellulose films with surface microstructures (M-CFs) can be fabricated via hot pressing and screen-template bonding. Using M-CF thin film as the triboelectric positive layer and nitrocellulose membrane as the triboelectric negative layer, C-TENGs are combined with excellent durability and signal stability (Fig. 8g) [153]. In addition, the measured voltage and current data can also be used to detect pulse changes and sound detection after human exercise (Fig. 8h), which is expected to be used as flexible self-powered wearable electronics. The introduction of −NO_3_ groups is one of the common and effective methods to rationally adjust the electrical polarity of CNFs. During the preparation of cellulosic materials, the CNF was treated with a mixed solution of HNO_3_ and H_2_SO_4_ to form a blended suspension. The suspension was sonicated and vacuum filtered through PVDF microporous membrane, flattened, and dried to prepare Nitro-CNF paper [155]. After CNF functionalization, the introduction of the nitrate group increases the tendency to gain electrons, and thus the Nitro-CNF paper acquires negative charges. Once physical contact is established between the nickel electrode and the Nitro-CNF paper, charge transfer is induced, thus providing new insights into the design and application of pressure sensors.

### Charge Density

When two different dielectric materials come into contact and separate from each other, static electricity is generated. In contact separation, more charges are transferred than what is assumed based on the charge densities from macroscopic tests [156]. The scope and extent of the application of TENGs in energy harvesting not only depend on the power density of the triboelectric materials, but they also have a quadratic positive correlation with the triboelectric charge density [157]. To increase the electrical output of TENGs, increasing efforts are being directed toward increasing their charge density. Charge excitation [158], physical surface engineering [132], and chemical functionalization [127, 159] have been studied to enhance the charge density of triboelectric materials. Coupling the tribolayer-surface and hysteresis-dielectric polarizations of ferroelectric materials can also achieve high triboelectric charge densities [160].

**Fig. 9:**
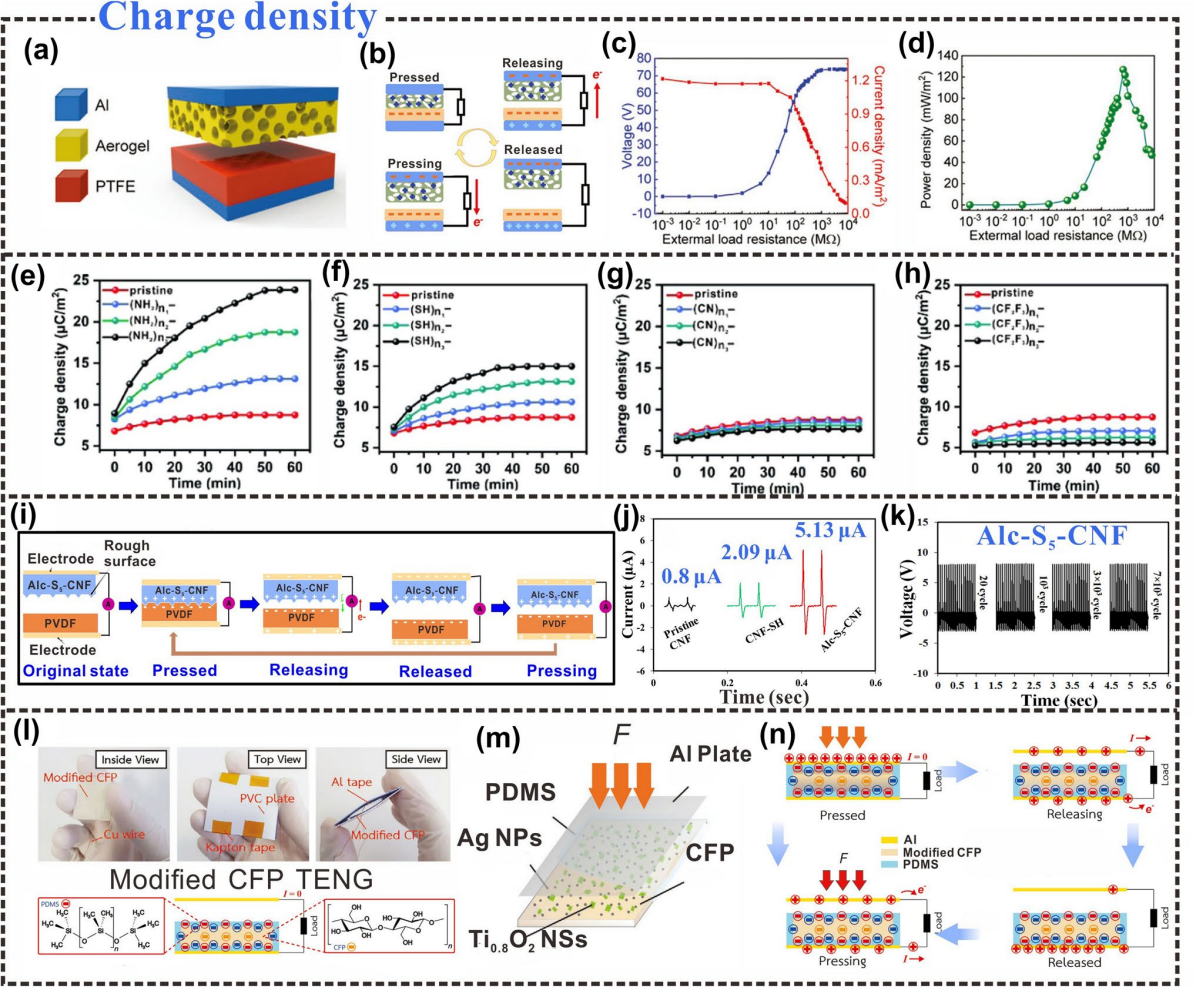
Charge density of cellulosic triboelectric materials. **a** Schematic diagram of the layer-by-layer cellulose II aerogel-based TENG structure. **b** The working mechanism of cellulose II aerogel-based TENG. **c** Current density and voltage output with different external load resistances. **d** Dependence of output peak power density on external load resistance. Reproduced with permission from Ref. [161]. Copyright 2020, Wiley–VCH. **e** The charge density of CNFs treated with different amounts of -NH_2_. **f** Different amounts of -SH treat the charge density of CNFs. **g** Different amounts of -CN manipulated the charge density of CNFs. **h** The charge density of different amounts of -CF_2_CF_3_ treatment. Reproduced with permission from Ref. [162]. Copyright 2021, Elsevier. **i** Schematic diagram of the working principle of Alc-S_5_-CNF/PVDF TENG device. **j** Comparison of triboelectric output currents among various TENGs made from pristine cellulose, CNF-SH, and Alc-S_5_-CNF films. **k** Cyclic durability test of Alc-S_5_-CNF/PVDF TENG. Reproduced with permission from Ref. [163]. Copyright 2020, Elsevier. **l, m** Modified CFP-TENG device, showing the structural design of the TENG device and photos from different angles. **n** Working mechanism of the modified CFP-TENG. Reproduced with permission from Ref. [164]. Copyright 2020, Wiley–VCH

There have been significant efforts toward improving the charge densities of C-TENGs. Natural cellulose (cellulose I) has been converted to regenerate cellulose (cellulose II) via dissolution and regeneration to prepare cellulose II aerogels. TENGs fabricated using cellulose II aerogels and PTFE films have been found to yield high electrical output characteristics (Fig. 9a) [161]. A uniform and continuous network structure is attributed to the cellulose II aerogel during the preparation process, and its unique nanostructure promotes charge accumulation. Cellulose II aerogels can not only induce charges on the contact surfaces but also distribute them across the surface of the structural network, such that the charge density increases with the charge distribution (Fig. 9b). An increasing charge density attributes a higher mechanical response sensitivity and electrical output to the highly porous cellulose II aerogel (Fig. 9c, d), demonstrating its potential for commercial applications.

As a two-dimensional material, phosphorene has attracted widespread interest owing to its high carrier mobility and good semiconducting properties [165]. Cui et al. fabricated a CNF/phosphorene TENG using the properties of phosphorene [166]. Nanocellulose was used to protect phosphorene from oxidation and as a substrate for the support body of the hybrid TENG. As charge capture sites, the charge-storage rate at the interface of the hybrid TENG was improved, increasing the surface-charge density and energy-collection performance. In the CNF/phosphorene hybrid system, a higher phosphorene content corresponded to a higher power output, and the power density of the CNF + 4P device was approximately 46 times that of the pure CNF device.

A change in the transferred charge originates mainly from a change in the chemical potential, which is determined by the functional group [167]. Amines (−NH_2_) are the most triboelectrically positive functional groups, which maximize charge density, while halogens (such as Cl) are the most negatively charged triboelectric functional groups [159, 168]. Recently, silane coupling agents with identical main chains but varying terminal functional groups have been used to regulate charge density ranges. The effect of various terminal functional groups on the charge density of cellulosic triboelectric materials was studied using 3-aminopropyltriethoxysilane (APTES), 3-mercaptopropyltriethoxysilane (MPTES), 3-cyanopropyltriethoxysilane (CPTES), triethoxy-1H, 1H, and 2H [162]. It was found that the surface potential increased as the number of -NH_2_ groups on CNF grafted increased. When CNFs with −NH_2_ and −SH functional groups are in contact with FEP, respectively, the transferred charges and surface charge density increase due to the tendency of the −NH_2_ and −SH functional groups to donate electrons (Fig. 9e, f). In contrast, the triboelectric output decreased after CF_2_CF_3_–CNFs and CN–CNFs were separated from FEP, respectively (Fig. 9g, h). This is because the CF_2_CF_3_- and CN-functional groups are electron-withdrawing groups and reduce the surface charge density. This also shows that by introducing different electron-withdrawing groups and electron-donating groups, the charge density on the surface of the material can be effectively adjusted. To improve the triboelectric charge density, Roy et al. achieved allicin–CNF conjugation or allicin grafting on CNFs using a novel thiol–ene click chemistry method (Fig. 9i) [163]. Owing to the triboelectric effect, the contact of two materials with stronger tribo-polarity resulted in a higher charge transfer and charge-density accumulation. The triboelectric output of the allicin–CNF-based TENGs was approximately 6.5 times that of the original C-TENGs. The triboelectric properties of cellulose were effectively improved (Fig. 9j), and the TENG exhibited sustained high-performance electrical output (Fig. 9k).

Dielectric modulation is often used to increase the charge density of triboelectric materials. Through dielectric modulation, the use of nanoparticles (such as BaTiO_3_ and Ag) can enhance the charge density of cellulosic materials. BaTiO_3_ nanoparticles and cellulose can be processed into porous aerogels via cross-linking, washing, and freeze-drying. Contact between the porous aerogel–paper and PDMS layers generates triboelectric charges with various electrical polarities on the surface of the material [129]. The enhancement of triboelectric output is mainly due to the enhancement of the charge-trapping ability and surface charge density of nanoparticles. The addition of Ti_0.8_O_2_ nanosheets/silver nanoparticles (Ag NPs) can generate more surface charge and potentially improve the performance of CNF-TENGs [169]. A multifunctional cellulose filter paper (CFP)-based TENG composed of dielectric Ti_0.8_O_2_ nanosheets (Ti_0.8_O_2_ NSs) and Ag NPs were fabricated using a simple dip-coating method (Fig. 9l) [164]. The addition of dielectric Ti_0.8_O_2_ NPs can effectively promote the generation of CFP surface charges, whereas the addition of Ag NPs provides a conductive path for charge transport (Fig. 9m). The dual modification of the multifunctional nanomaterials can improve the charge density and output performance of the device (Fig. 9n).

## Rational Design of Advanced Cellulosic Triboelectric Materials for Self-powered Wearable Electronics

**Fig. 10:**
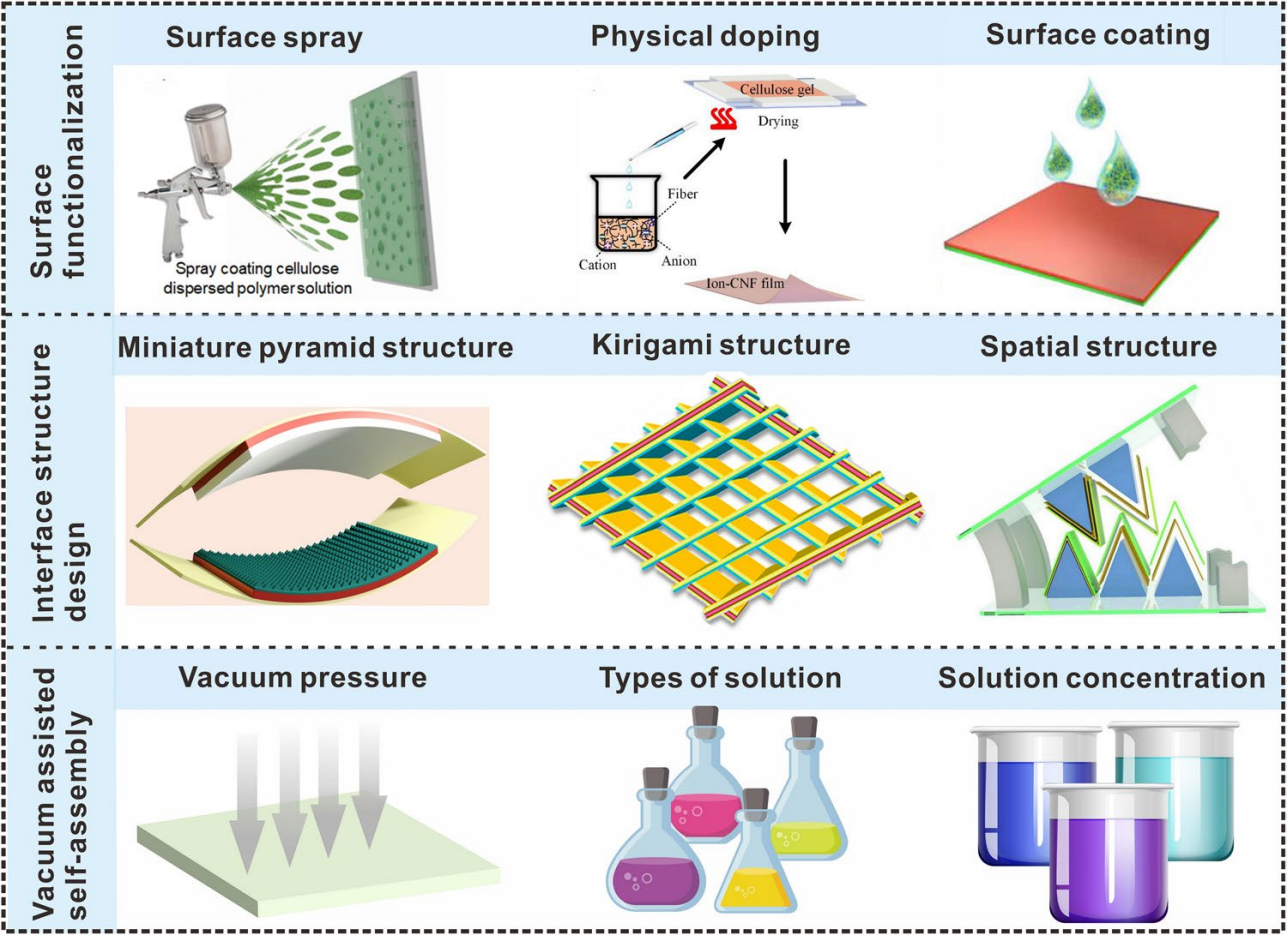
Rational design strategies of cellulosic triboelectric materials. Reproduced with permission from Ref. [172], Copyright 2020, American Chemical Society; Ref. [173], Copyright 2020, American Chemical Society; Ref. [132], Copyright 2018, Elsevier; Ref. [174], Copyright 2022, Elsevier; Ref. [175], Copyright 2017, American Chemical Society; Ref. [176], Copyright 2019, Elsevier

Reasonable regulation of triboelectric properties and fine control of the geometric structure of triboelectric materials are of great significance for the preparation of triboelectric materials used in specific environments [170, 171]. In recent years, the types of cellulosic triboelectric materials used in the field of self-powered wearable sensors have gradually increased, and material design methods need to correspond to the application of materials. This section mainly summarizes the rational design strategies of cellulosic triboelectric materials, including surface functionalization, interfacial structure control, vacuum-assisted self-assembly, etc. These strategies provide new perspectives for designing cellulosic self-powered wearable electronics for different application scenarios (Fig. 10).

### Surface Functionalization

**Fig. 11:**
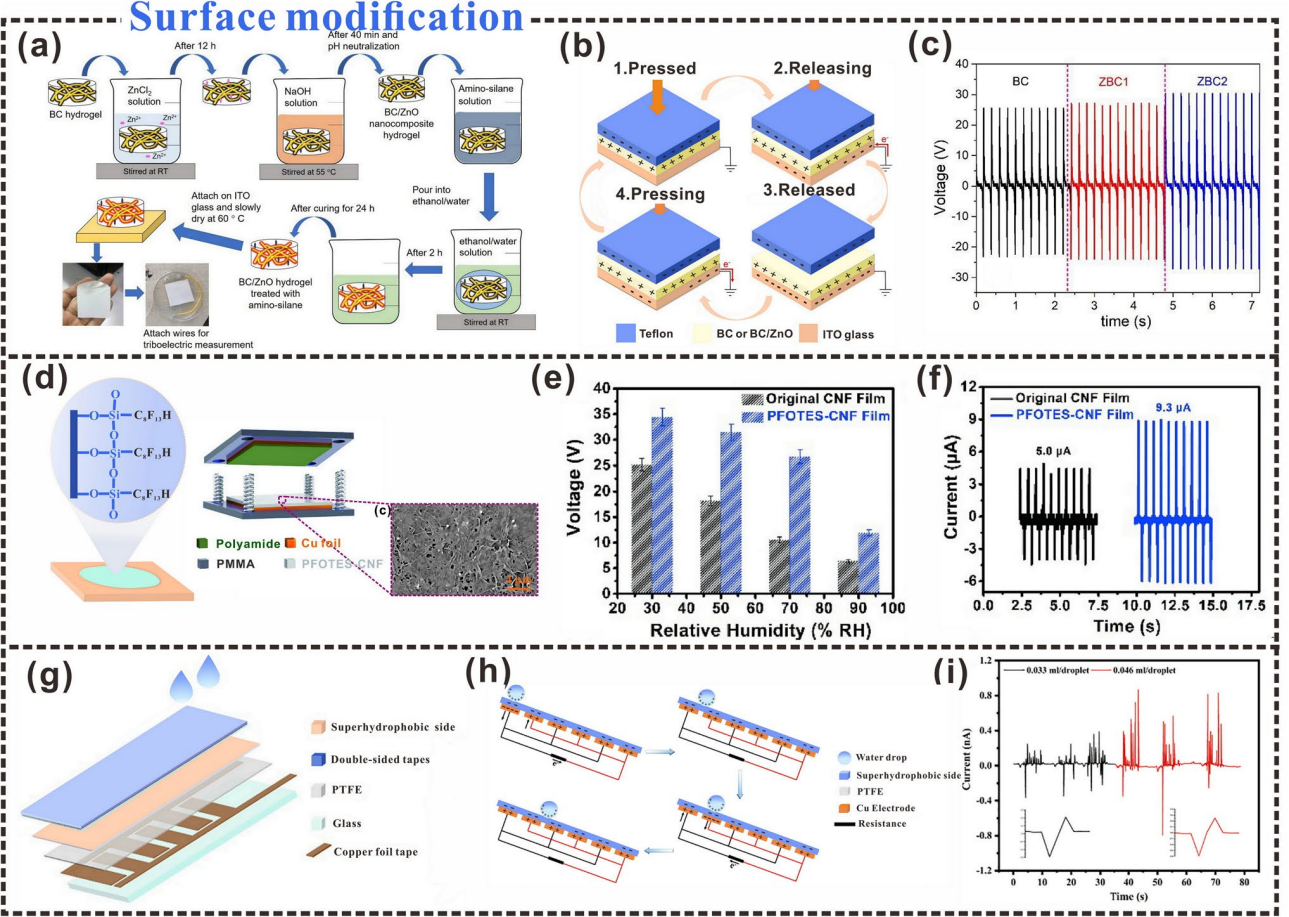
Surface functional design strategies. **a** The manufacturing process of BC/ZnO (ZBC). **b** Energy generation process using vertical contact separation mode with a single electrode. **c** The voltage of BC, ZBC1, and ZBC2 at 5 Hz. Reproduced with permission from Ref. [179]. Copyright 2020, American Chemical Society. **d** Surface modification and schematic diagram of TENG structure. **e** Open circuit voltage of TENG. **f** Short-circuit current of PFOTES-CNF thin film-based TENG. Reproduced with permission from Ref. [178]. Copyright 2021, Elsevier. **g** Schematic diagram of the structure of H-TENG. **h** The working mechanism of H-TENG. **i** Output currents of H-TENG generated by water droplets of different volumes at the same drop rate. Reproduced with permission from Ref. [180]. Copyright 2021, Elsevier

Cellulosic functional materials are being increasingly applied in the fields of energy harvesting and storage and environmental protection. However, the preparation of cellulosic materials with multiple functionalities remains a challenge. Surface functionalization is a good approach for the preparation of multifunctional cellulosic materials. In energy harvesting, contact electrification of triboelectric materials is the main energy source, and surface functionalization can be regarded as an effective design idea for cellulosic triboelectric materials for sensing [176, 177]. In addition, the strategy of surface functionalization can also enhance the triboelectric properties of cellulosic TENG [178]. Aminosilane functionalization of BC/ZnO (ZBC) nanocomposites can improve the adhesion between thin films and ITO substrates (Fig. 11a) [179]. The enhancement of triboelectric charge transfer is mainly attributed to the rough surface of the ZnO nanoparticles. During the contact electrification process, there are obvious triboelectric signals (Fig. 11b, c), which indicate that the adhesion between the BC/ZnO film and the ITO substrate has been improved, and energy harvesting can be performed sensitively. The functional endowment of cellulosic materials can be achieved via not only physical doping but also graft copolymerization, in-situ polymerization, and impregnation methods. Table 2 shows the effects of different modification methods on the triboelectric properties. Nie et al. [178], developed a method to prepare bio-based TENG through grafting fluorine-containing functional groups onto the surface of natural cellulose (Fig. 11d), which not only improved the triboelectric output of TENG but also increased the durability of electronic devices. The cellulose films were chemically modified using triethoxy-1*H*,1*H*,2*H*,2*H*-tridecafluoro-*n*-octylsilane (PFOTES) to form PFOTES-CNFs. TENGs prepared from PFOTES-CNF and polyamide materials exhibited significantly high open-circuit voltages and short-circuit currents (Fig. 11e, f). Grafting fluorine-bearing silane chains onto the surface of CNFs increased the polarity of the tribological layers and improved the hydrophobicity of the CNF films. The contact angle of the modified CNF films was increased from 52° to 128°. Thus, the modified TENG components were attributed to excellent moisture resistance and stability, and their open-circuit voltage and short-circuit current retention could be maintained at approximately 70%, even under conditions of 70% humidity.

**Table 2:** Effects of different modification methods on triboelectric properties

Triboelectric material design method	Production process	Triboelectric output	Advantages	References
Spraying process	Spraying cellulose and polyvinyl alcohol solution on a conductive flexible substrate	Voltage: 50 V Current: 5 μA	Eliminate mechanical interference; Efficient collection of mechanical energy	[172]
Lution dipping method	Cationic modification of the surface of the wood	Voltage: 335 V Current: 9.74 μA	Eliminate the brittleness of wood and improve the surface charge density of wood	[181]
Ntroduce functional groups	The amino group is introduced into CNF	Voltage: 195 V Current: 13.4 μA	Increase the surface polarizability of cellulosic materials	[182]
Spray deposition	Hydrophobic modification of cellulose paper	Voltage: 165 V Current: 60 μA	Excellent resistance to water, oil, and dust	[183]
Synthesis of composite materials	The cationic nanocellulose and C_3_N_4_ were introduced into polyacrylamide (PAM)	Voltage: 0.5 V Power density: 0.18 W m^−2^	High toughness, high elasticity	[66]
Complexation bindings	Preparation of MXene Ti_3_C_2_Tx/CMC airgel with CMC as cross-linking agent	Voltage: 54.73 V Current: 1.22 μA Power density: 0.40294 W m^−2^	Improve mechanical properties and exhibit good flexibility	[184]
Electrospinning	Filling the pores of the CNF-PVA fiber mat (eCPFM) with CNF suspension	Voltage: 78.5 mV Current: 553.7 nA	Higher yield strength, tensile strength, elongation at break, and toughness	[185]
TEMPO oxidation	Conversion of CNF to COOH-CNF using TEMPO oxidation to obtain TOCNs	Power density: 0.96 W m^−2^	Geometrically, chemically and electrostatically asymmetric	[186]
Water dispersion method	TOCN/MoS_2_ composite thin films were prepared	Voltage: 4.1 V Current: 0.21 μA	Good mechanical properties, tensile strength, and elongation at break	[187]
Dissolve, crosslink, soak	Soak cross-linked cellulose in glycerol/NaCl/H_2_O solution for cryopreservation	Voltage: 120 V Current: 0.95 μA Charge: 30 nC Power density: 0.315 W m^−2^	Good robustness, flexibility, transparency, and conductivity	[131]
Improve surface functional groups	Remove impurities by centrifugation, add cross-linking agent	Power density: 1.273 W m^−2^	Enhanced dielectric constant	[188]
Functional group grafting	The cellulose fibers were treated with epichlorohydrin and ethylenediamine	Voltage: 147 V Current: 4.9 μA Power density: 0.1378 W m^−2^	Change the surface polarity of the cellulose fibers	[189]
Amino groups were introduced onto the cellulose	Chemical modification of cellulose surface by aminopropyl triethoxy silane	Voltage: 160 V Current: 10 μA	Enhance the electrostatic induction effect	[190]

The surface functionalization of triboelectric materials can also be used for harvesting liquid energy. For example, multifunctional and superhydrophobic CCP was prepared via in-situ polymerization of polypyrrole on the opposite surface of the paper, and concurrently with a PDMS/candle-soot-treated MXene coating [180]. Cellulose composite paper (CCP) with superhydrophobic properties can be used as a hydro triboelectric nanogenerator (H-TENG) to harvest water energy (Fig. 11g). The water droplets generate triboelectric charges with the air or drifting particles during the falling and rolling process (Fig. 11h), and the single-pulse output current of the two electrodes reaches a maximum of 0.8 μA (Fig. 11i), which increases the triboelectric output of the H-TENG.

Superhydrophobicity is a prerequisite for liquid–solid TENGs in harvesting liquid energy [191]. A suspension of nano-fumed silica and epoxy resin was obtained under magnetic stirring and sonication. The suspension was sprayed on the surface of the filter paper by a spray gun, and the sprayed filter paper was soaked in hydrolyzed triethoxy-1H, 1H, 2H, 2H-tridecafluoro-n-octylsilane (PFOTES) to obtain a superhydrophobic film, which was successfully applied in self-powered raindrop energy harvesting [192]. D-TENG was prepared by the superhydrophobic membrane. After the water droplets landed on the surface of the superhydrophobic cellulose paper, the superhydrophobic cellulose paper contact with the polytetrafluoroethylene (PTFE) membrane and the amount of charge transfer also increased with the increase of the droplet volume and the falling height. In addition to spraying the modified solution onto the surface of the cellulose film for surface functionalization, a triboelectric film was also prepared by spraying a cellulose and poly(vinyl alcohol) solution onto the conductive substrate [172]. The preparation of C-TENGs using surface-functionalized cellulose films is the key to the application of cellulosic materials in harvesting liquid energy. The strategy of surface functionalization provides a new idea to increase biocompatible materials used in self-powered wearable sensors in special environments (e.g., rainy, snowy, foggy).

### Interfacial Structural Control

The improvement of triboelectric output is not only related to the material but also related to the structure of the material surface. It is worth noting that the structural design of triboelectric materials is also considered as an effective way to solve energy supply and sensing problems [193]. For example, the triboelectric output of cellulosic materials can be enhanced by constructing microns or nanopatterns of pyramids, squares, or hemispheres on their surfaces [194]. In addition, nanoparticles [195], microsurface structuring [196], and other methods can improve the effective contact area and promote the triboelectric effect. Consequently, more triboelectric charges are generated on the positive and negative triboelectric layers, increasing the triboelectric output [197].

**Fig. 12:**
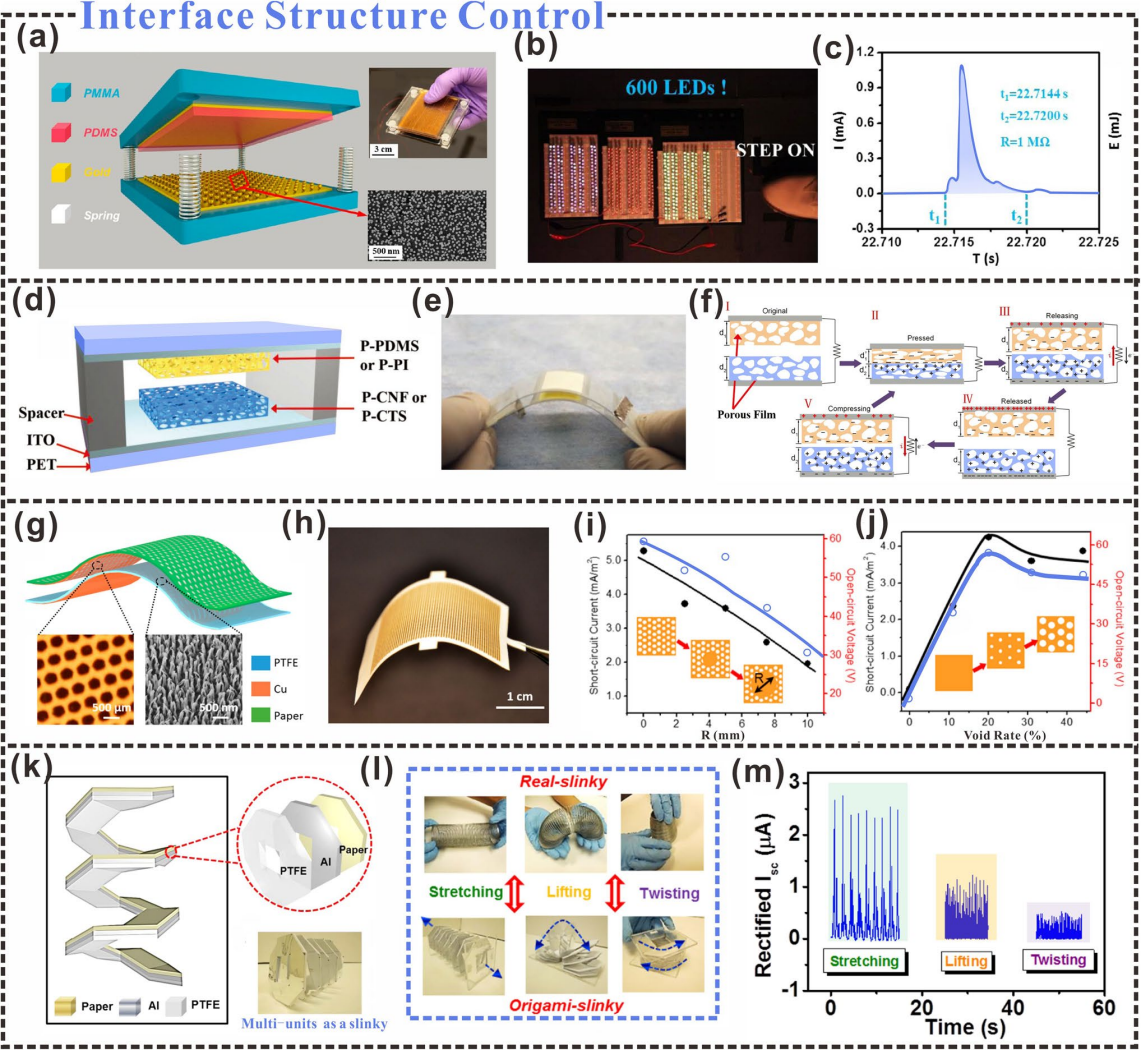
Interfacial structural control design strategies. **a** Nanoparticle-based triboelectric generators. **b** The photo of a nanoparticle-attached triboelectric nanogenerator driving an LED. **c** The current pulse output is generated when a human foot touches a 1 MΩ load resistance. Reproduced with permission from Ref. [206]. Copyright 2019, Elsevier. **d** Schematic diagram of the highly porous aerogel film TENG (A-NG). **e** The photo of TENG (A-NG). **f** Schematic diagram of the working principle of TENG (A-NG). Reproduced with permission from Ref. [207]. Copyright 2018, American Chemical Society. **g** Preparation of porous paper with porous design-TENG. **h** The photo of the porous paper electrode. **i** The effect of central hole distribution on electrical output. **j** The effect of the void-to-surface ratio on the electrical output of the device. Reproduced with permission from Ref. [208]. Copyright 2015, American Chemical Society. **k** Structure and photographs of a slinky TENG. **l** TENG realizes multi-angle motion through a spring. **m** Multi-angle energy harvesting. Reproduced with permission from Ref. [205]. Copyright 2015, American Chemical Society

Common techniques, such as lithography [198], nanoimprint lithography [199], and laser interference lithography [200, 201], are effective methods for building surface microstructures. Table 3 shows the effects of different structural design options on the triboelectric output. Micro/nanoscale cone and micro bowl structures can be uniformly and efficiently fabricated on the surfaces of Cu and PDMS thin films using laser scanning ablation (Fig. 12a), which can also significantly increase the effective contact area and surface roughness of the triboelectric layer surface [202]. The use of the laser-ablated Cu and PDMS films as the triboelectric positive and negative layers, respectively, generates more triboelectric charges during the contact process and a larger dipole moment, thus, improving the triboelectric output. Stepping on the TENG can power 600 LEDs (Fig. 12b), and a current pulse output of 1.5 mA can be generated by bringing it in contact with a load resistance of 1 MΩ (Fig. 12c).

**Table 3:** Effects of different interface structures on triboelectric properties

Structural shape	Triboelectric output	Advantage	Design method	References
Termite nest-like porous structure	Voltage: 478 V	Increase charge accumulation	Polymer blending method	[126]
Two-dimensional CMFs/CNFs/Ag hierarchical nanostructures	Voltage: 21.9 V Current: 0.17 µA Charge: 8.3 nC	High air pollutant removal efficiency	Deposition method	[203]
Porous Nanocomposite fabric (PNF)	Voltage: 448 V Power density: 0.25 W m^−2^	Good mechanical strength, deformability, flexibility, and washability	Dry casting method	[59]
CNF/rabbit hair composite porous aerogel	Voltage: 110 V Current: 11.3 μA Power Density: 3.4 W m^−2^	High energy harvesting efficiency and high sensitivity	Freeze drying method	[79]
Kirigami structure	–	Lightweight high charge output, and ultra-portable	Laser cutting machine, and electron beam sink product method	[175]
3D micro/nano hierarchically patterned structure	Voltage: 55.8 V Current: 0.94 μA Power Density: 0.029 W m^−2^	Improve the triboelectric response	All-printing method	[138]
Multilayer porous cellulose template	Voltage: 84 V Current: 0.94 μA	Increase the surface area of the sheet and increases the active sites and micro-capacitor network	Depressurized impregnation	[204]
Dense porous structure	Power Density: 1.237 W m^−2^	Have good stability	Physical doping	[188]
Microstructured surfaces	Voltage: 97 V Current: 5.7 μA	Enhance electrical performance	Hot-pressing drying combined with the screen mesh templates	[153]
Wrinkled structure	Voltage: 55 V Current: 14 μA	Increase the triboelectric charge density	Dry-creping	[205]

Microstructural dimensions can be constructed not only by constructing cellulose films using sophisticated photolithography but also by using sandpaper. Zhang et al. [209], developed an embossing process based on sandpaper to micro/nanotextured the surface of tribological pairs (paper card and Teflon tape). During the sample preparation process, micro/nano patterns of uneven sand grains on the surface of the sandpaper were quickly transferred to the surface of the triboelectric layer. The triboelectric output exhibited regular variation with the number of micro/nanostructures on the corresponding sandpaper-grain surface. The triboelectric output of the sandpaper first increased and then decreased in the particle-size range of 60–800. When the grit of the sandpaper was 120, the output voltage reached its peak value. In addition, the electrostatic forces were distributed unevenly on the micro/nanotextured surface, making it possible to control the direction of the surface droplet movement. The micro/nanotextured surface made it easier for the electrostatic force to exhibit an asymmetrical distribution, and the droplets on the surface could be effectively controlled. The controllability of droplets presents strong application potential in various industries, such as portable inkjet printing sensing systems, biomedical sensing micro/nano systems, and other fields.

The surface microstructure is one of the key parameters affecting the output performance of TENGs. Microscale patterns can be obtained on the surface of the cellulose fiber layer by sanding [206]. The nylon fiber layer and the polypropylene fiber layer with polytetrafluoroethylene are hot-pressed. After the hot pressing, the two layers are placed together on the single layer of the cellulose fiber layer to construct the TENG structure. Punching holes in the cellulose fiber layer can help to complete the charge transfer in the vertical contact separation mode. This method can be effectively used for human body motion sensing and has the potential to be used as flexible wearable electronics. An increase in porosity can provide more space surface for the TENG and generate more surface charge on the porous space surface [210]. Taking the aerogels prepared from highly porous CNF and chitosan (CTS) as an example, the highly porous CNF and CTS aerogels (P-CNF and P-CTS) were used as triboelectric positive materials and porous polyethylene Siloxane paired to assemble P-CNF/P-PDMS and P-CTS/P-PDMS paired A-NGs (Fig. 12d, e). The high performance and stability of A-NGs show potential applications in the development of self-powered wearable electronics. When the triboelectric positive and negative electrodes were in contact, the presence of the microstructure promoted charge transfer efficiency, which generated additional charges on the porous surface [211]. The triboelectric output and the porosity of the aerogel were found to be positively correlated (Fig. 12f) [212].

Xing et al. innovatively employed arrays of microholes for acoustic response enhancement. A TENG for harvesting acoustic energy was fabricated using paper and PTFE films as the positive and negative triboelectric layers, respectively (Fig. 12g) [213]. The area of the central nonporous region and pore size was found to be closely related to the triboelectric output. With an increase in the central nonporous area, the maximum peak output decreased (Fig. 12h), which was mainly attributed to a weakened membrane vibration due to the air-dumping effect of the hole-free part. The triboelectric output decreases with increasing film pore size (Fig. 12i), which is caused by a decrease in the contact area (Fig. 12j). The concept and design presented in this work can be extensively applied to a variety of other circumstances for either energy-harvesting or sensing purposes, for example, self-powered wearable electronics, military surveillance, jet engine noise reduction, the low-cost implantable human ear, and wireless technology applications.

In addition to constructing different micro-nanostructures on the surface to enhance the triboelectric output, more and more people use the establishment of spatial structures to prepare triboelectric nanogenerators from the perspective of macroscopic interfaces [207]. Dong et al. [45], fabricated all-yarn-based self-charging power textiles with an interesting weft-knitting technique with spatially knitted structures, which are highly elastic, flexible, and stretchable. The knitting TENG fabric can generate electric energy with a maximum instantaneous peak power density of 85 mW m^−2^ and light up at least 124 light-emitting diodes. Assembled woven kinetic textiles can sustainably drive wearable electronic devices (such as calculators or thermo-hygrometers) with the energy generated by human motion. A TENG has been constructed by attaching Ag particles to the surfaces of modified CNFs and combining them with a 3D gear-like structure [176]. The three-dimensional space structure increased the frictional contact area by approximately four times that of an ordinary planar structure and the three-dimensional gear-like structure provided more contact area for the TENG. The amount of triboelectric charge and output increased accordingly, and the open-circuit voltage and short-circuit current increased by 109% and 40%, respectively. Recently, paper-based origami structures have been extensively studied in the construction of spatial structures, and pendulum-like paper-based zigzag multilayered hybrid TENGs have been used for solar, tidal, and mechanical energy harvesting [208]. The physical contact between two different triboelectric materials creates a triboelectric charge on the surface, and the relative separation of mechanical motion results in a potential drop between the two electrodes. The triboelectric output is boosted by the flow of electrons between the two electrodes, which provides a new perspective for applications such as intelligent identification and sensory response.

The triboelectric contact area can be increased via an origami structure, and the wiring process of the triboelectric nanogenerator can be simplified. The paper substrate, PTFE film, and aluminum foil can be joined into multiple octagonal units using the origami folding process to construct a TENG (Fig. 12k). The adjacent octagonal units can be easily connected via paper joints without any additional wiring processes (Fig. 12l) [214]. The structure is used as a portable pressure sensor that can harvest mechanical energy from all directions. The structure can be used as a pressure sensor to harvest mechanical energy in all directions (Fig. 12m). Kirigami has also been recently used in the study of triboelectric nanogenerators, and Wu et al. fabricated a highly stretchable TENG using traditional kirigami techniques [215]. The prepared kirigami-TENG (K-TENG) had a higher triboelectric output. When periodic tension was applied to it, the electrical signal produced a cyclic AC output on the load between the electrode and the ground. In addition, K-TENG structures also have interlocking linear and rectangular kirigami patterns, which can induce charge separation through the expansion and contraction of the kirigami structure, thereby effectively generating a triboelectric output. The device is subtly applied to self-powered wearable acceleration sensing and self-powered book open and close sense. Guo et al. fabricated a self-charging power supply unit (PC-SCPU) using ultralight kirigami [175]. The self-charging power supply unit consisted of a TENG (PC-TENG) and a paper-based supercapacitor (P-SC). By applying periodic pressure to the PC-TENG, an AC output was achieved, and the rectified power could be stored directly using the P-SC, which is a feasible way to prepare portable energy harvesting and storage electronic devices. Control of interface structure can effectively enhance the surface-charge transfer efficiency of cellulosic triboelectric materials, thereby enhancing the triboelectric output. Thus cellulosic triboelectric materials can be widely applied in various fields.

### Vacuum-Assisted Self-Assembly

**Fig. 13:**
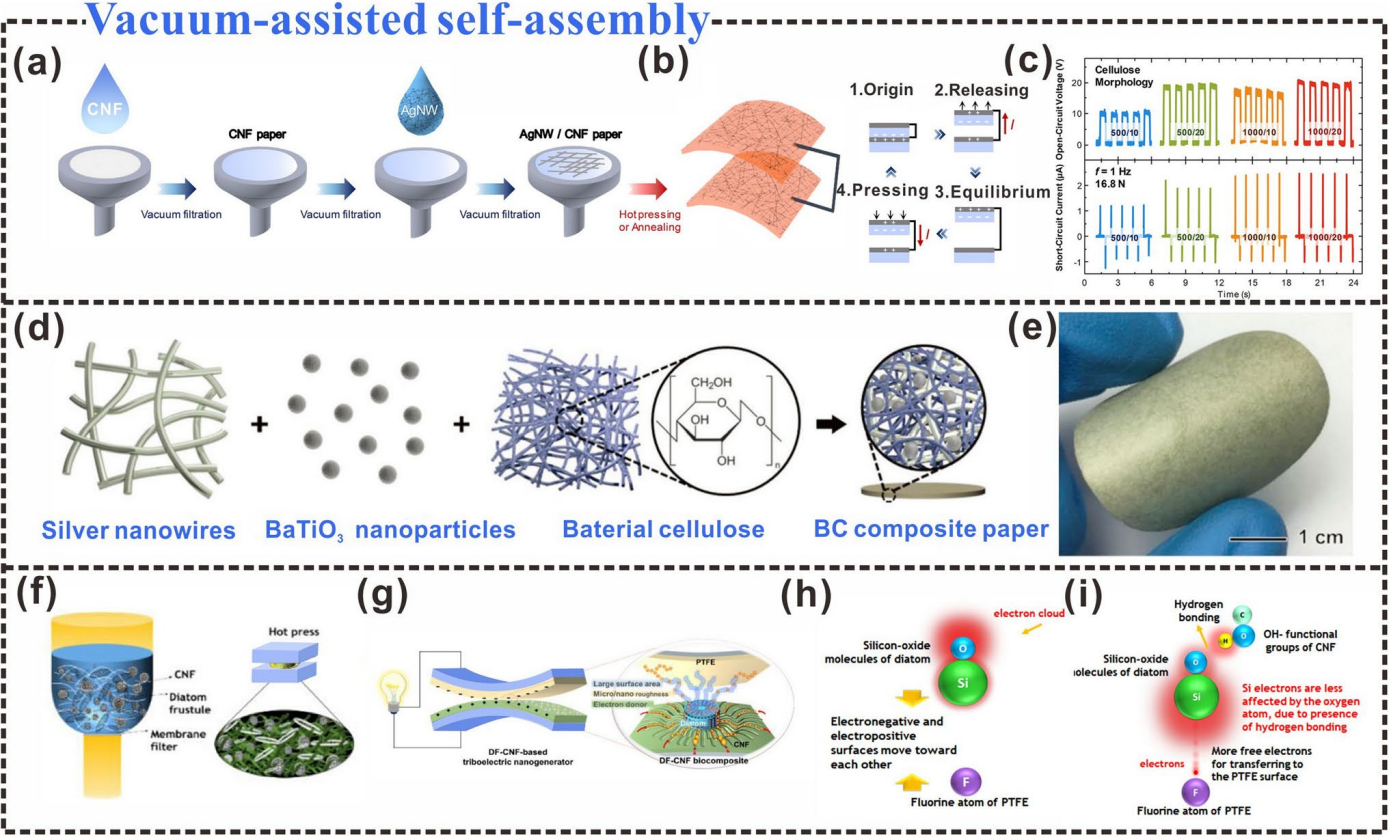
Vacuum-assisted self-assembly design strategies. **a** Schematic illustration of AgNWs/CNF paper fabricated using vacuum filtration technique. **b** Schematic diagram of the structure of AgNWs/CNF-TENG and its operating mechanism. **c** The triboelectric output (open-circuit voltage and short-circuit current) of TENG depends on the cellulose morphology. Reproduced with permission from Ref. [218]. Copyright 2018, Elsevier. **d** Schematic diagram of the fabrication of highly conductive ferroelectric BC composite paper prepared by vacuum directional induction. **e** The photo of BC composite paper. Reproduced with permission from Ref. [26]. Copyright 2019, Elsevier. **f** DF-CNF composites were prepared by vacuum orientation induction and hot pressing. **g** DF-TENG with biocompatibility. **h, i** Polarity of hydrogen bonds and interatomic bonds, results of hydrogen bonds between DF and CNF. Reproduced with permission from Ref. [219]. Copyright 2020, American Chemical Society

Vacuum-assisted self-assembly provides another fast and simple process for fabricating CNF-based TENGs. An AgNW/CNF paper was fabricated using a CNF suspension and silver (AgNW) solution via vacuum-assisted self-assembly (Fig. 13a) [216]. The amount and concentration of the AgNW solution were adjusted via vacuum-assisted self-assembly to replace time-consuming deposition methods such as electrode coating. The amount and concentration of AgNWs solution can be flexibly adjusted during the vacuum-assisted self-assembly process to replace time-consuming deposition methods such as coating electrodes. The AgNWs/CNF paper acts as a self-contained film, allowing electrodes and insulators to come into contact without a substrate (Fig. 13b). The effective contact area is positively correlated with the triboelectric output, and the larger the effective contact area is, the greater the triboelectric output of the TENG will be. And different cellulose states also have a certain effect on the triboelectric output (Fig. 13c). The effectiveness of the vacuum-assisted assembly depended on not only the amount and concentration of the solution but also the number of filtrations and the applied pressure. The increase in filtration times and applied pressure resulted in a smaller average diameter of CNFs, which makes the surface morphology of the CNF film more uniform, and the effective contact area of the CNF paper increases, accompanied by the increase of the triboelectric output. The AgNWs/CNF paper not only plays a role in harvesting environmental energy, but also can be used in folding sensors, humidity sensors, and has flexibility and portability. In addition, preventing particle exposure is another way to increase the effective contact area, and vacuum-assisted self-assembly can reduce particle exposure during film formation. The high-dielectric-constant BaTiO_3_ particles were encapsulated in the BC matrix via vacuum-assisted self-assembly (Fig. 13d) [26], which prevented particle exposure and afforded a higher effective contact area on the surface of the tribological layers (Fig. 13e), consequently enhancing the output of the triboelectric layer [217].

Vacuum-assisted self-assembly can assist materials to better combine. The mixture suspension of bacterial cellulose (BC), AgNW, and barium carbonate inorganic nanoparticles (BTO NP) were prepared by vacuum-assisted self-assembly to prepare cellulosic composite paper, and the cellulosic composite paper and PTFE film were used to construct BC-TENG. With the assistance of the vacuum-assisted self-assembly method, the ferroelectric material is evenly distributed on the surface of the composite paper, so that the aligned dipoles in the BTO domain can be positively polarized with better quality. As a result, a greater charge transfer between the BC composite paper and the PTFE surface was obtained, resulting in a higher triboelectric output. The BC-TENG can also be used as a self-powered wearable sensor, such as a pulse sensor, heart sensor, and blood pressure sensor [220]. A eucalyptus-pulp CNF suspension was mixed with diatoms (DF) via vacuum-assisted self-assembly and hot-pressing to prepare composite membranes with diatoms as tribo-bio-additives (Fig. 13f) [221]. Diatoms formed 3D structures on the surface of the composites via vacuum-assisted self-assembly. Introducing micro/nano patterns on the surface and increasing its roughness improved the triboelectric output power (Fig. 13g). When DF was added to CNF, diatom bio-silica (SiO_2_) frustules were in interaction with CNF through hydrogen bonding, resulting in a tighter association of diatoms with CNF (Fig. 13h, i). Therefore, the cellulosic triboelectric material has a stable triboelectric signal output during contact separation. To explore the effect of film thickness on the triboelectric output of cellulosic triboelectric materials, CNF-DF films with different thicknesses were prepared via vacuum-assisted self-assembly. The CNF-DF film thickness increased from 15 to 60 μm, and the triboelectric output was improved. However, when the thickness increases beyond 60 μm, the voltage and current show a sharp decrease trend. It demonstrates that when the dielectric layer is thin, the charges generated by contact charging are weakened and the charges trapped on the CNF surface are reduced. Taking advantage of this feature, the CNF-DF was prepared as a self-powered smart mask for human health monitoring. Improving the performance of triboelectric materials not only provides a promising strategy for exploring high-performance wearable power sources but also shows great application potential in portable electronics and e-textiles [218]. Therefore, a combination of multiple strategies to enhance the triboelectric output of triboelectric materials is a good research idea. The vacuum orientation-induced silanization of cellulose was used to improve the positive polarity and enhance the charge transfer between materials [182]. Vacuum-assisted self-assembly gives more flexibility to the material preparation process, not only to control the concentration and amount of the solution but also to obtain more transfer charges by increasing the effective contact area.

## Emerging Applications of Cellulosic Self-powered Wearable Electronics

With the advent of the Internet of Everything era, the demand for wearable electronics has increased, and the running time and stable output have gradually become requirements for wearable electronics. As mentioned earlier, rational design strategies such as surface functionalization, interface structure design, and vacuum-assisted self-assembly have a significant impact on the triboelectric properties of cellulose, which can not only be used to tune the surface charge properties of materials, but also increase the charge density of triboelectric materials. Other properties such as flexibility, sensing properties, and durability are given to the cellulosic material through the modulation of design strategies. With these advantages, it has been widely used in the field of wearable electronic products such as the induction detection of human motion [222], the preparation of hand tactile sensors [223], the monitoring of human physiological signals [219] the enhancement of human–machine contact [224] and the early warning of fire [225].

### Energy Harvesting

With the advent of the wireless networking era, there is a growing need for self-powered wearable, portable bioelectronics [226]. These bioelectronic products can sense and harvest energy from human motion and biomechanical vibrations [227]. The intermittent energy generated by human movement is considered a rich source of energy. The body is rich in energy from walking, movement of joint parts, and stretching of muscles [228]. The microstructure triboelectric film developed by combining microcrystalline cellulose (MCC) and biocompatible polyvinyl alcohol (PVA) was used to prepare a button battery-type TENG [130]. The fabricated button cell-type TENG is placed under the medial arch of the human foot for efficient energy harvesting from daily human activities such as walking, running, and jumping. In addition, in the energy harvesting of human body movement, the triboelectric charge capture ability of the modified CNF can be increased by the sulfonic acid functional group acting as a deep trap, which can effectively improve the triboelectric output, thereby realizing wearable biomechanical energy harvesting [229]. Dip coating is also one of the effective ways to effectively enhance the surface potential of cellulosic triboelectric materials [230]. By controlling the dipping time, the surface potential and output performance can be effectively controlled. It provides a good research base for cellulosic TENG to be used as self-powered energy harvesting and human motion detection. In the study of biomechanical energy harvesting, bacterial cellulose also provides an effective idea for self-powered motion sensing. A triboelectric generator (TEG) made of porous polyvinylidene fluoride (PVDF) and bacterial cellulose (BC) layers was successfully used as a self-powered motion sensor [231].

**Fig. 14:**
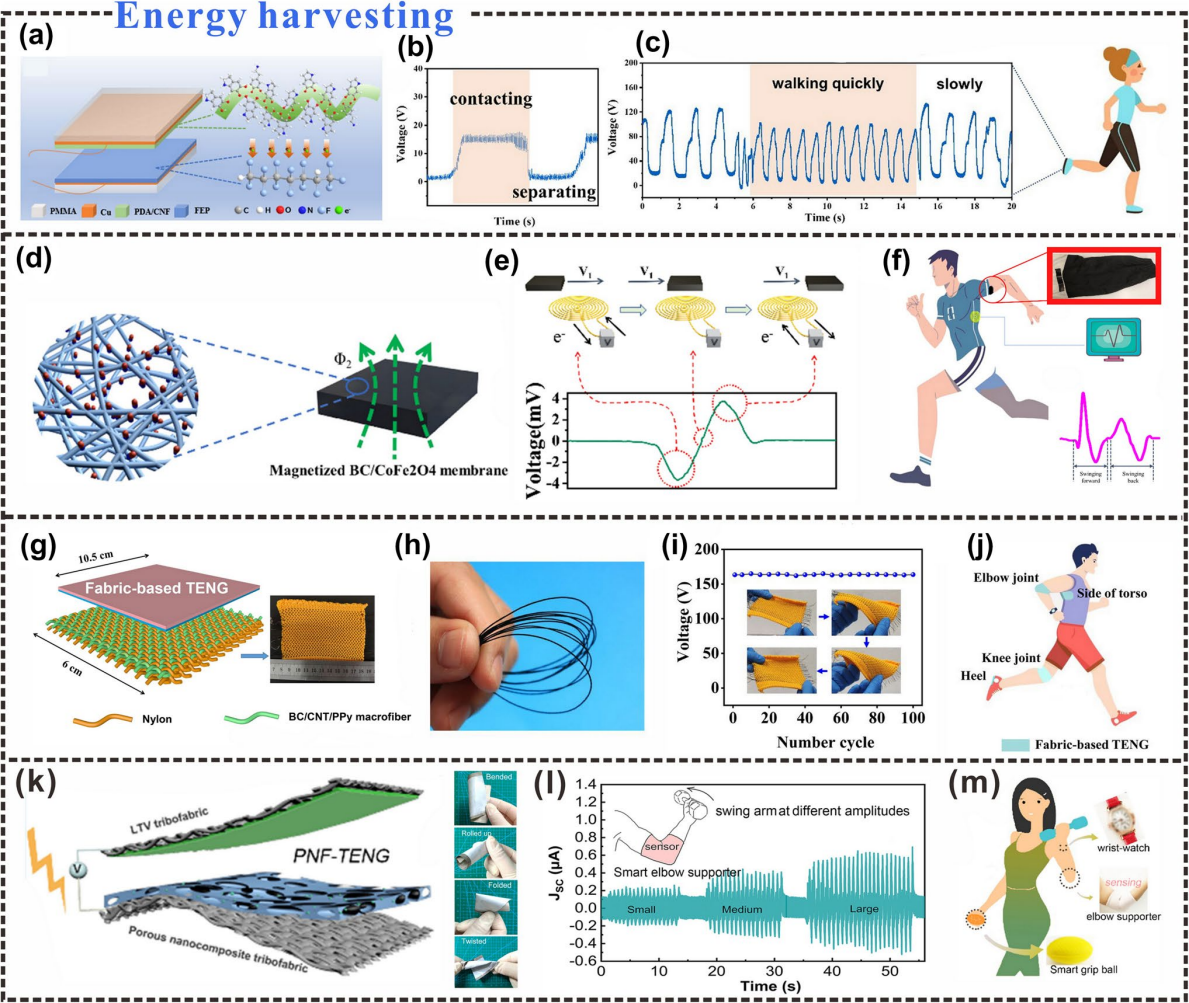
Self-powered wearable sensors for energy harvesting. **a** Electrode structure and electron transport of PDA/CNF-TENGs. **b** Voltage output during contact and separation. **c** Voltage output while walking. Reproduced with permission from Ref. [236]. Copyright 2022, Springer Nature. **d** Composite magnetic film of CoFe_2_O_4_ nanoparticles. **e** Schematic diagram of the induced voltage in one cycle. **f** Schematic diagram of the smart jacket and its application. Reproduced with permission from Ref. [237]. Copyright 2022, Elsevier. **g** Schematic diagram of the fabric-based TENG structure. **h** Cellulosic conductive coarse fibers. **i** Output voltage of the fabric-based TENG under 100 mechanical deformation cycles. **j** Photograph of the TENG of the fabric as self-powered motion sensing. Reproduced with permission from Ref. [25]. Copyright 2022, Springer Nature. **k** Fabric TENG is fabricated for wearable fabric sensors. **l** Smart sports elbow support. **m** Fabric TENG assisted in rehabilitation training. Reproduced with permission from Ref. [59]. Copyright 2020, American Chemical Society

The recognition of human movement states such as walking, falling, and running can be easily recognized by sensors [232]. Wearable-TENGs have recently output electrical signals in a self-powered manner to detect human motion [233]. Cellulosic triboelectric nanogenerators (TENGs) can power various monitoring devices in an environmentally friendly and sustainable manner. CNF is combined with polydopamine through chemical functionalization. By adjusting the electron affinity of the surface functional groups of CNF, the surface potential difference between the triboelectric positive layer and the triboelectric negative layer can be effectively increased, and the surface charge density can be increased [234]. An environmentally friendly high-performance triboelectric nanogenerator based on polydopamine/cellulose nanofiber (PDA/CNF) composite film and fluorinated ethylene propylene was fabricated for human motion energy harvesting and self-powered motion monitoring (Fig. 14a) [235]. PDA/CNF-TENGs do not require an external power source for energy and sense in a self-powered manner. It can successfully identify multiple motion states, such as changes in the electrical signal of the sensor under pressure or relaxation (Fig. 14b). More importantly, in the process of human walking, the difference between fast walking and slow walking can be detected sensitively (Fig. 14c), with stable, repeatable, and recoverable signals. The combination of polydopamine and cellulosic materials has inspired more application ideas for biomass materials in the field of wearable electronic sensors. Lin et al. [58], introduced octadecylamine (ODA) and polydopamine (PDA) onto cellulose paper by Schiff base reaction to produce hydrophobic cellulose paper (HCP). Owing to the amino group, hydrophobicity, and rough surface, hydrophobic cellulose papers exhibit excellent triboelectric properties and stability. Combining the stretchable lantern structure with hydrophobic cellulose paper, L-HCPTENG is obtained for human motion and self-powered sensing, which can not only harvest energy from motion but also monitor hand flapping, walking, and running status.

For the preparation requirements of wearable sensors, high comfort is necessary for wearable devices, where flexibility and portability become the signature features. CoFe_2_O_4_ nanoparticles and bacterial cellulose (BC) can be combined to form a multilayer flexible magnetic film by in situ co-precipitation combined with hot pressing to solve the problems of flexibility and convenience in wearable devices (Fig. 14d) [238]. The facile interface optimization method not only improves triboelectric performance but also enhances its application potential in wearable flexible sensors by combining multilayer flexible magnetic films by hot pressing. The flexible magnetic film goes through two stages of passing through and returning to the entire copper coil, forming a complete motion cycle, during which an AC voltage output is generated (Fig. 14e). Taking advantage of this feature, a smart jacket was prepared based on a flexible magnetic film, which can monitor human motion signals by detecting the swing speed and frequency. The smart jacket has a reference value for motion pattern recognition and can be successfully applied to human motion monitoring (Fig. 14f).

Based on the pursuit of the flexibility of wearable sensors, fabrics have become an important material source for wearable electronic devices. Smart fabrics have recently shown promising applications in the field of efficient energy harvesting [239]. Hu et al. [25], prepared cellulosic conductive thick fibers by wet stretching and wet twisting bacterial cellulose hydrogel mixed with carbon nanotubes and polypyrrole. Using cellulosic conductive thick fibers for weaving, a fabric-based triboelectric nanogenerator (TENG) was designed to efficiently drive commercial electronics (Fig. 14g). The fabric-based TENG has superior environmental friendliness, mainly because the cellulosic conductive thick fibers constituting the fabric-TENG can be quickly dissolved in the cellulase solution (Fig. 14h). And the output voltage can remain stable at 160 V after 100 mechanical deformation cycles (Fig. 14i). The fabric-TENG can effectively harvest energy from human motions such as walking, running, jumping, arm raising, arm bending, and leg raising for real-time sensing (Fig. 14j). It has potential application value in athlete training, fitness exercise and physical rehabilitation of patients. Efficient and reliable wearable sensors are the embodiment of wear resistance and equipment durability. Fabrics with strong charge accumulation (PNF) have been fabricated for use in wearable fabric sensors [59]. The PNF was prepared by dry casting method using cellulose acetate and Al_2_O_3_ fillers, which is an effective triboelectric cathode material (Fig. 14k). Triboelectric nanogenerators fabricated from PNF can serve as wearable power sources and self-powered sensors. Based on PNF-TENG, an intelligent motion elbow support was designed and manufactured to detect the swing range of the arm during barbell movement (Fig. 14l). Even in the smart grip ball, real-time monitoring of grip strength can be contribute to with rehabilitation training (Fig. 14m). Cellulosic wearable electronics have a wide range of applications in biomechanical energy harvesting and motion sensing, and have great potential for development in the future.

### Tactile Sensing

**Fig. 15:**
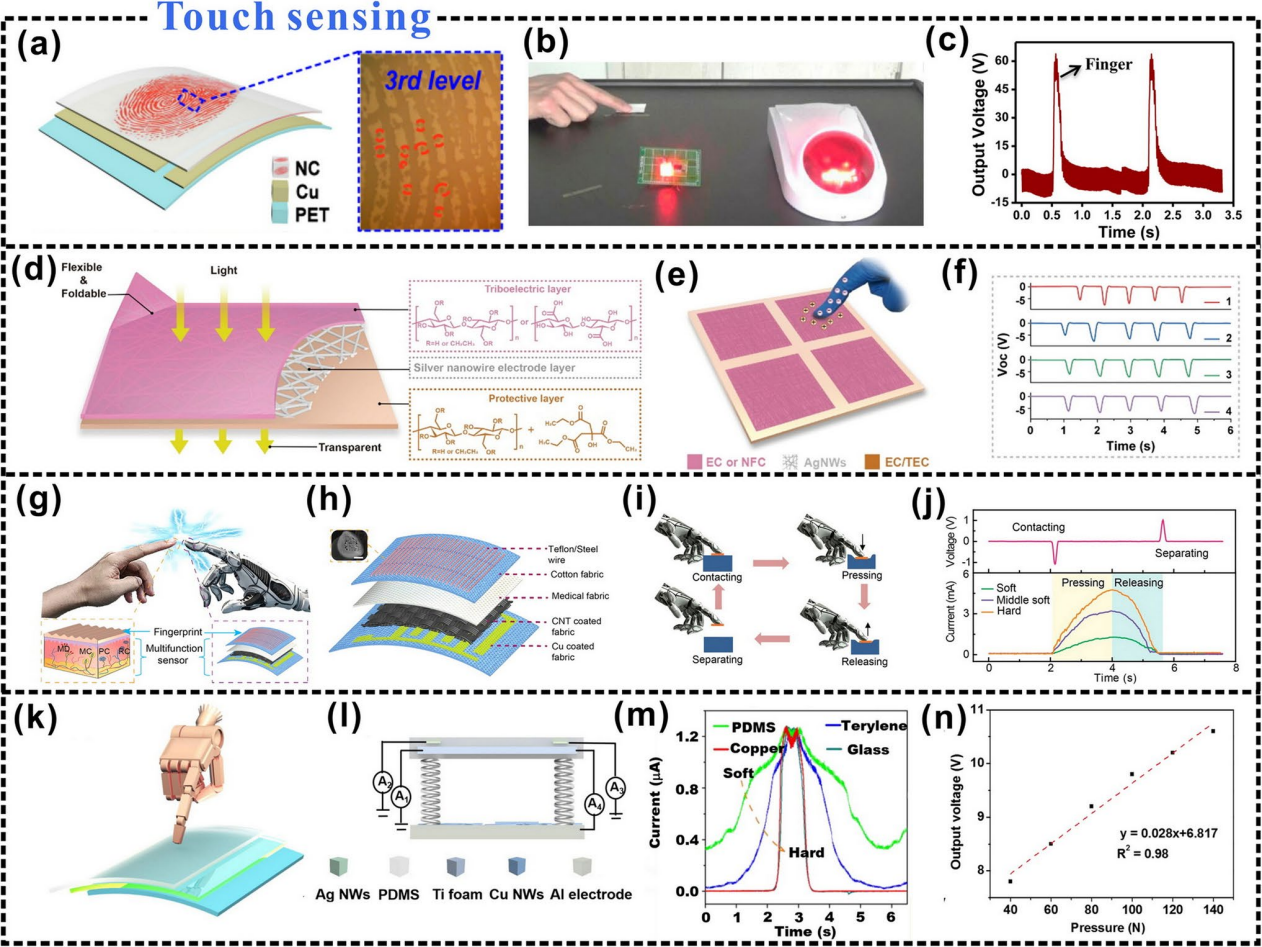
Wearable self-powered sensors for tactile sensing. **a** Monolithic triboelectric nanosensor composed of nitrocellulose membrane. **b** Monolithic triboelectric nanosensor for fingerprint detection. **c** Provides sharp tactile sensing under the touch of a finger. Reproduced with permission from Ref. [243]. Copyright 2016, American Chemical Society. **d** Cellulosic multilayer film (CMLF). **e** The cellulosic multilayer film is used as a tactile password switch. **f** Cellulosic multilayer TENG with high sensitivity. Reproduced with permission from Ref. [60]. Copyright 2022, Elsevier. **g** Cotton fabric tactile sensor. **h** Composition of the tactile sensor. **i** Contact separation process of the tactile sensor. **j** Real-time feedback of the relative hardness of the object. Reproduced with permission from Ref. [244]. Copyright 2022, Elsevier. **k** Dual-mode TENG for tactile sensors. **l** Composition of the dual-mode TENG. **m** Short-circuit current output of different materials under dual-mode TENG. **n** The triboelectric signal increases with the force applied on the dual-mode TENG. Reproduced with permission from Ref. [61]. Copyright 2017, American Chemical Society

With the development of the smart industry, the Internet of Things (IoT) is distributed in the development of various functional sensors and interactive interfaces [64]. Tactile interaction interfaces are crucial for the interaction of wearable electronic devices and intelligent robots with the environment and humans and have important impacts in the fields of intelligent mechanical sensing [240], precision digital control [241], smart home systems [236], and advanced industrial manufacturing [237]. With the promotion of environmental protection, more and more green and pollution-free wearable electronic devices are being used in real life, such as the development of security systems. Yang et al. [242], prepared nitrocellulose membranes (NC) based on the principle that nitrate groups can interact with strong dipoles of peptide bonds in proteins. Nitrocellulose membrane and polyethylene terephthalate (PET) film constitute a monolithic triboelectric nanosensor (Fig. 15a). With their excellent sensitivity, monolithic triboelectric nanosensors have been successfully used in self-powered alarm systems to detect fingerprints through self-triggering (Fig. 15b). The self-powered alarm system triggers distinct triboelectric signals under the touch of a finger, providing keen touch detection (Fig. 15c).

Different from the traditional complex intelligent mechanical sensing, the tactile sensor has gradually attracted the attention of the public because of its simplicity and lightness. For the development of secure systems, password identification is also a top priority. Various patterns, shapes, and structures can be formed by origami or kirigami techniques to prepare cellulosic multilayer films (CMLF) (Fig. 15d) [60]. The film is flexible and foldable, can be easily cut or folded into various spatial structures, and can be used in password identification systems. TENGs derived from cellulosic multilayer films can be used as touch password switches (Fig. 15e). When an experimenter wearing nitrile rubber gloves touched the TENG with a finger, the surface of the EC layer lost electrons and generated a triboelectric signal (Fig. 15f), with high sensitivity. The combination of CMLF electronic equipment and artificial intelligence has the potential to contribute to the fields of smart home systems, concealed intelligent monitoring, wireless control, and wearable sensing.

The development of soft robots is of great significance to the development of the artificial intelligence industry. Wearable tactile sensors have recently been applied to the development of soft robots, so the materials and working modes of tactile sensors have received more attention. Many polymers are currently used in tactile sensors, such as polydimethylsiloxane (PDMS), polyethylene terephthalate (PET), polyimide, and other polymer substrates, which often lead to problems of lack of breathability and discomfort [245]. Compared with polymers, textiles are one of the ideal materials for wearable electronics because of their strength, softness, and breathability in terms of human comfort [246]. Pang et al. [247], developed tactile sensors using cotton fabrics as substrates that can easily perform health monitoring or intelligent perception of complex surfaces for soft robots (Fig. 15g). The tactile sensor consists of two sensing layers (Fig. 15h). When the tactile sensor is in contact with or separated from the touching object, the TENG sensing layer will generate instantaneous negative and positive voltage signals due to contact electrification, respectively (Fig. 15i). The detection of the relative hardness of different objects can also provide effective real-time feedback (Fig. 15j), which significantly promotes the development of wearable sensing devices and soft robots.

To improve the ability of soft robots to perceive and interpret the environment, a dual-mode TENG was fabricated for tactile sensing (Fig. 15k) [61]. The bimodal TENG consists of polydimethylsiloxane (PDMS) on top and titanium foam inside (Fig. 15l). Once the different materials contact with the PDMS film, a triboelectric signal is generated. In addition to common natural polymers (cellulose), metals (copper foil), nylon, polymethyl methacrylate (PMMA), and inorganic materials (soda lime glass) have been shown to obtain output currents from dual-mode TENG through small forces (Fig. 15m). As the force applied on the dual-mode-TENG increases, the triboelectric signal gradually increases (Fig. 15n). The dual-mode TENG can simultaneously detect and quantify data such as tactile sensation, stiffness, object position, and pressure. It has the potential to be used in areas such as mobile technology, computers and electronic skins.

### Health Monitoring

**Fig. 16:**
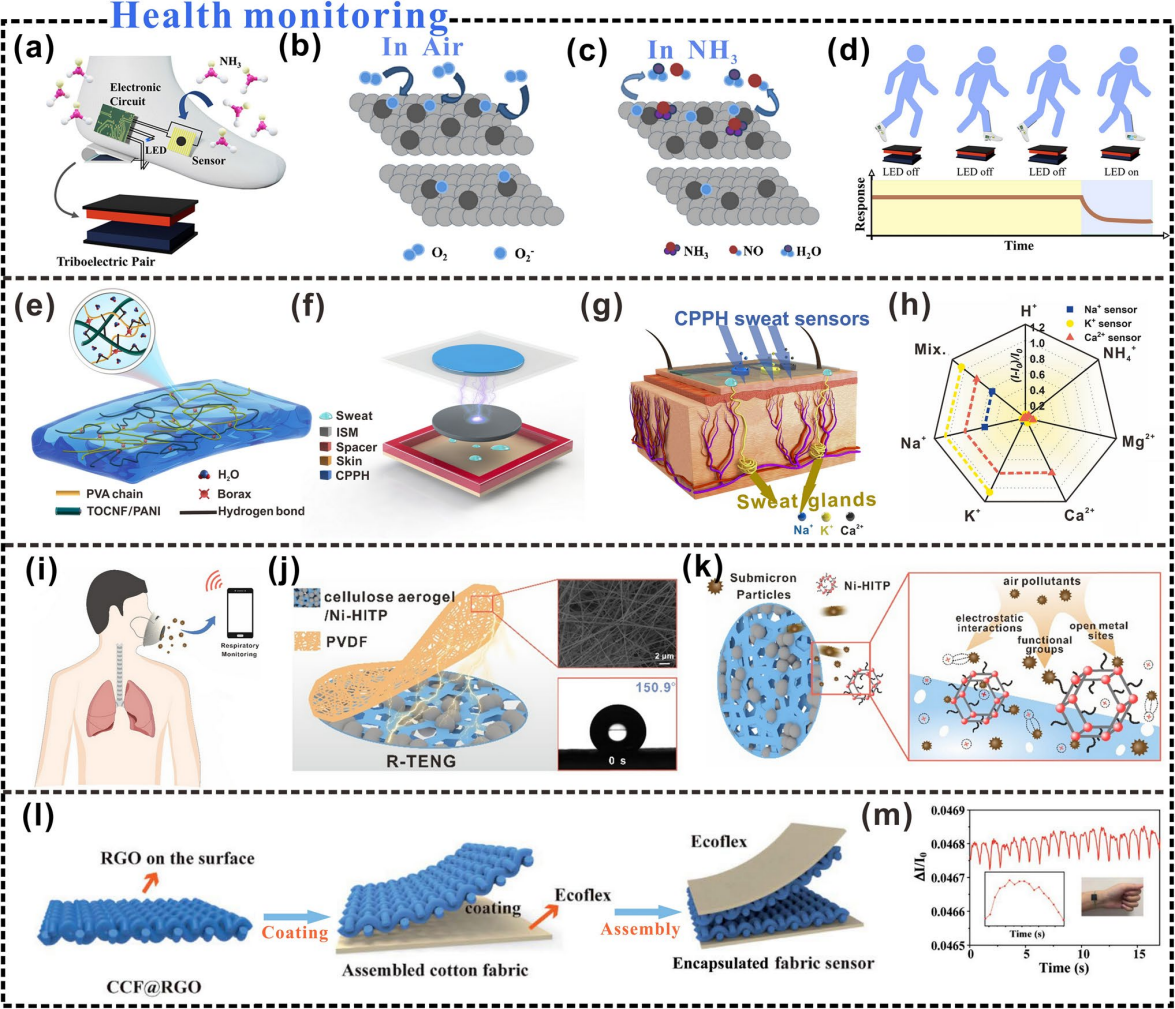
Self-powered wearable sensors for health monitoring. **a** The sensor of MXene/TiO_2_/C-NFs heterojunction. **b, c** Schematic diagram of the gas mechanism of MXene/TiO_2_/C-NFs. **d** Visualized NH_3_ monitoring system. Reproduced with permission from Ref. [256]. Copyright 2022, American Chemical Society. **e** Cellulosic conductive hydrogel. **f** Cellulosic conductive hydrogels are used as electrodes. **g** Cellulosic conductive hydrogel as a sweat sensor. **h** The sweat sensor can detect the composition of human sweat in real-time. Reproduced with permission from Ref. [62]. Copyright 2022, Wiley–VCH. **i** Self-powered filter assembled from cellulose aerogel (CA). **j** Cellulose aerogels with abundant three-dimensional micro-nanopores. **k** The self-powered filter has the function of filtering and adsorbing submicron particles. Reproduced with permission from Ref. [257]. Copyright 2022, Elsevier. **l** Schematic illustration of the fabric sensor based on CCF@RGO. **m** Signal response for human pulse monitoring. Reproduced with permission from Ref. [258]. Copyright 2020, Royal Society of Chemistry

Wearable sensors have made great progress in providing non-implantable solutions for health monitoring, early diagnosis, and disease management. But the main obstacle in the field is the inability to accurately and consistently collect data from the human body [248]. Therefore, it is imminent to explore novel materials, sensing technologies, and integration strategies to fully realize the potential of flexible electronics in wearable sensing. Cellulose has excellent segment modification and biocompatibility, and its non-toxic and harmless characteristics make them have a strong advantage in monitoring human health [243]. At present, the common types of cellulosic materials are mainly films [249], hydrogels [244, 250], aerogels [251], and fabrics [252]. Real-time monitoring of hazardous gases is important for identifying environmental components that are hazardous to human health, and sensors based on MXene/TiO_2_/C-NFs heterojunctions have been fabricated for NH_3_ identification (Fig. 16a) [253]. The MXene/TiO_2_/C-NFs heterojunction sensor is a TENG composed of MXene/cellulose acetate NFs (MXene/CA-NFs) thin films. Among them, the synergistic effect of MXene and titanium dioxide (TiO_2_) enhances the performance of the MXene/TiO_2_/C-NFs heterojunction sensor (Fig. 16b, c), so sensing sensitivity is guaranteed accordingly. When the NH_3_ concentration is in the range of 1–100 ppm, the heterojunction sensor can make a sensitive linear response. Finally, a monitoring system was assembled from the TENG self-powered sensor, equivalent circuit, and LED visualizer to detect the leakage of NH_3_ (Fig. 16d). In addition to this, gas-sensitive materials can be prepared by improving the adsorption of cellulosic materials to improve the monitoring ability of NH_3_ [68]. The removal of particulate matter (PMx) for atmospheric pollution can also be achieved by self-powered supply [254, 255]. Using self-powered sensing to remove and detect harmful gases can not only reduce the dependence on external power sources but also can avoid possible health hazards.

Human sweat composition is critical for physiological health assessment. At present, a cellulosic conductive hydrogel electrode made of polyaniline and polyvinyl alcohol/borax in situ polymerization cross-linked has been fabricated for self-powered sweat sensors (Fig. 16e) [62]. Among them, ion-selective membrane (ISM) was used as triboelectric positive material, polydimethylsiloxane (PDMS) was used as triboelectric negative material, and cellulosic conductive hydrogel was used as an electrode (Fig. 16f). The ISM is separated from the PDMS by positive and negative contacts to detect Na^+^, K^+^, and Ca^2+^ concentrations in real-time (Fig. 16g) with high selectivity and sensitivity in the normal ion concentration range (Fig. 16h). It has a wide range of applications in health monitoring and wearable artificial intelligence. To improve the accuracy of preventive healthcare and medical diagnosis, Zheng et al. developed a smart insole system for foot behavior monitoring [259]. The smart insole system successfully monitors gait changes in real-time with high sensitivity and excellent durability, which provides a new perspective for self-powered wearable devices for gait monitoring.

Atmospheric pollution caused by particulate matter has recently become a major impact on human life. Therefore, the development of a simpler and more portable self-powered healthcare product is imminent. Fu et al. [260], prepared conductive CA/Ni-HITP composites by in situ growing Ni-HITP on cellulose aerogel (CA) and combined with PVDF film to fabricate R-TENG. A wearable self-powered air filter was assembled by R-TENG (Fig. 16i). As shown in Fig. 16j, the cellulose aerogel has abundant three-dimensional micro-nano-scale pores, and the pores are uniformly filled by Ni-HITP. Among them, CA/Ni-HITP is used as a positive triboelectric material and electrode, and PVDF is used as a negative triboelectric material, which plays the role of filtering and adsorbing submicron particles during the contact electrification process (Fig. 16k). For the degradation of PM particles, Zou et al. [203], proposed an alternative scheme. Using CMFs/CNFs as a template, a self-powered cellulose fiber-based triboelectric nanogenerator (cf-TENG) system is proposed. The cf-TENG system can remove PM_2.5_ with an efficiency of up to 98.83%, and can monitor the respiratory status without an external power supply. A novel and sustainable strategy for self-powered wearable electronics in healthcare applications is presented.

Textile-based wearables are commonly found in health monitoring devices. Single-electrode triboelectric CCF@RGO fabrics were prepared by coating polydimethylsiloxane (PDMS) on the surface of cotton fabrics (Fig. 16l) [256]. The space between the stacked CCF@RGO and the conductive properties make it a strong candidate for flexible pressure sensors. Due to the high sensitivity and superior flexibility of the CCF@RGO fabric sensor, the textile sensor can be successfully used in human motion and health monitoring, such as the detection of joint bending ability and pulse monitoring (Fig. 16m).

### Human–Machine Interaction

**Fig. 17:**
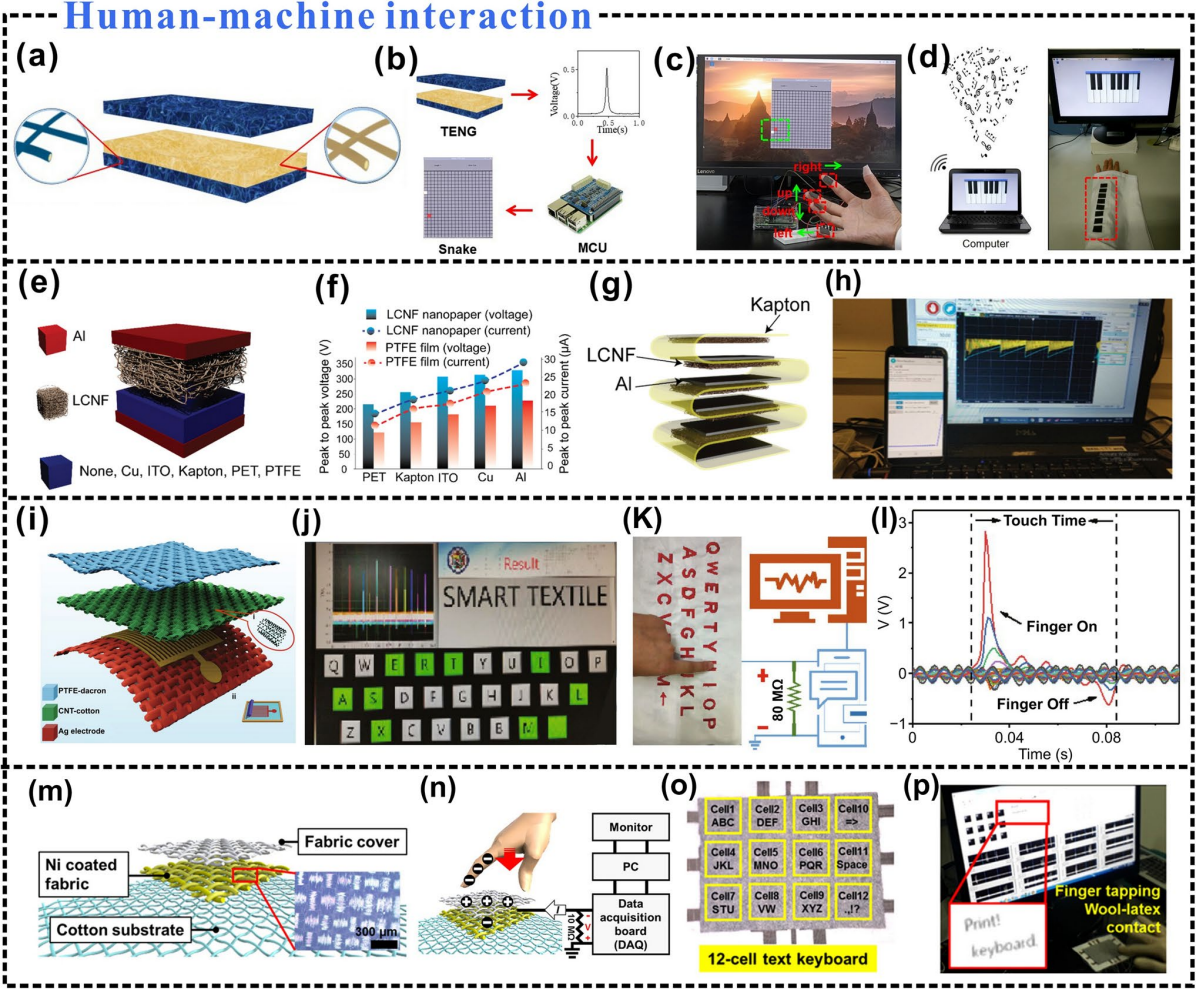
Self-powered wearable sensors for human–machine interaction. **a** TENG based on BC-CNT-PPy/BC/BC-CNT-PPy sandwich structure. **b** All-cellulose energy harvesting and human–computer interaction devices. **c** The all-cellulose TENG activates the “snake” software. **d** All-cellulose TENG is used as a wearable interactive interface to control an electronic piano. Reproduced with permission Ref. [23]. Copyright 2022, Elsevier. **e** Triboelectric nanogenerator of lignocellulose nanofibers (LCNFs). **f** Triboelectric data of LCNF and PTFE after contact separation. **g** Cascaded TENG. **h** Cascaded TENG power nodes running wireless communication. Reproduced with permission from Ref. [264]. Copyright 2022, Elsevier. **i** F-TENG composed of CNT/cotton conductive fabric. **j** F-TENG is used to detect external keystrokes. **k** Circuit composition of the measuring system. **l** Electrical signals generated by keystrokes at specific locations. Reproduced with permission from Ref. [265]. Copyright 2021, Springer Nature. **m** All-fabric TENG self-powered wearable keyboard. **n** Triboelectric signal generated by a self-powered wearable fabric sensor during contact separation. **o** 12-cell keyboard. **p** Typing under a different number of strokes and stroke units. Reproduced with permission from Ref. [266]. Copyright 2018, Elsevier

With the development of machine learning and artificial intelligence technology, future human–computer interaction will become more intelligent, and it is expected to provide more humanized services [261]. The wearable triboelectric nanogenerators provide interesting incorporation points for realizing natural, intuitive, and real-time human–computer interactions [262]. Zhang et al. [23], developed a TENG based on the sandwich structure of BC-CNT-PPy/BC/BC-CNT-PPy (Fig. 17a), which is an all-cellulosic energy harvesting and human–computer interaction device with biodegradable properties (Fig. 17b). The mechanical energy harvested by the whole cellulose TENG can also be used as a switch signal recognized by a single-chip microcomputer, triggering a processing circuit and an embedded software program. In this way, the all-cellulose TENG can be used as a wearable interface to control/trigger some software-level (or hardware-level) applications, such as the “snake” software that can be triggered by finger touch (Fig. 17c). The all-cellulose TENG was successfully used to power commercial electronic devices and was used as a wearable interface to control an electronic piano (Fig. 17d). Environmentally friendly materials have gradually assumed a more important role in wearable electronic devices, which contribute to the further development of wearable electronic devices. Roy et al. [263], converted cellulose acetate from waste cigarette filters into a high-performance triboelectric nanogenerator (TENG). The TENG obtained from the preparation was connected to the watch and showed good practicality. It has significant implications for eco-friendly electronics, bio-adaptive human–machine interfaces, and smart biomimetic functional devices.

Because pure cellulose exhibits weak intrinsic triboelectric positive properties, cellulosic materials have rarely been studied as triboelectric layers [257]. However, in the process of development of sustainable HCI sensors, Tanguy et al. proposed to make triboelectric nanogenerators based on lignocellulose nanofibers (LCNF) by retaining lignin from the raw material (Fig. 17e) [258]. After comparing the triboelectric data of common triboelectric positive electrode materials with LCNF and PTFE respectively, it was found that LCNF nano paper has stronger triboelectric negativity than PTFE film (Fig. 17f). When LCNF nano paper as a triboelectric negative layer and aluminum as a triboelectric positive layer are combined in a zigzag structure to form a cascaded TENG (Fig. 17g), it can be used as a power node for running wireless communication (Fig. 17h). The cascaded TENG can maintain efficient signal transmission to realize a self-powered wireless monitoring system for AI technology or IoT.

Owing to the poor conformability of traditional polymers, flexible and stretchable textiles combined with self-powered sensors are considered as new insights into wearable electronics in the IoT era. The triboelectric positive layer is made of CNT/cotton conductive fabric by layer-by-layer self-assembly method, while the triboelectric negative layer is prepared by immersing polyester cloth in 60% polytetrafluoroethylene (PTFE) solution, and the positive and negative triboelectric layers are combined to form F-TENG (Fig. 17i) [267]. The F-TENG is high-voltage sensitive and can generate electrical signals to detect external keystrokes (Fig. 17j). Each electrical signal output terminal of the measurement system is connected to a separate keyboard and acquisition card, which can reduce the impact of environmental noise (Fig. 17k). When the keystroke position of a specific position starts, a peak output voltage up to 2.8 V is obtained from the corresponding channel, while the signal from other keys is less than 1.2 V, so as to distinguish the position where the keyboard is struck (Fig. 17l). Among the various components for building e-textiles, wearable devices such as keyboards are one of the most important components for human–computer interaction. The all-fabric-based TENG array as a touch-sensing component demonstrates a self-powered wearable keyboard (Fig. 17m) [268]. The self-powered wearable fabric sensor can generate corresponding triboelectric signals during touch and release (Fig. 17n). As shown in Fig. 17o, alphabets are assigned to the 12-cell keyboard. By tapping different times and different cells, the word "keyboard" can be successfully displayed on the computer (Fig. 17p). Considering the textile industry-friendly process and materials, cost-effectiveness, and feasibility, textile sensors are expected to be strong candidates for wearable devices in the IoT era.

### Intelligent Fire Warning

**Fig. 18:**
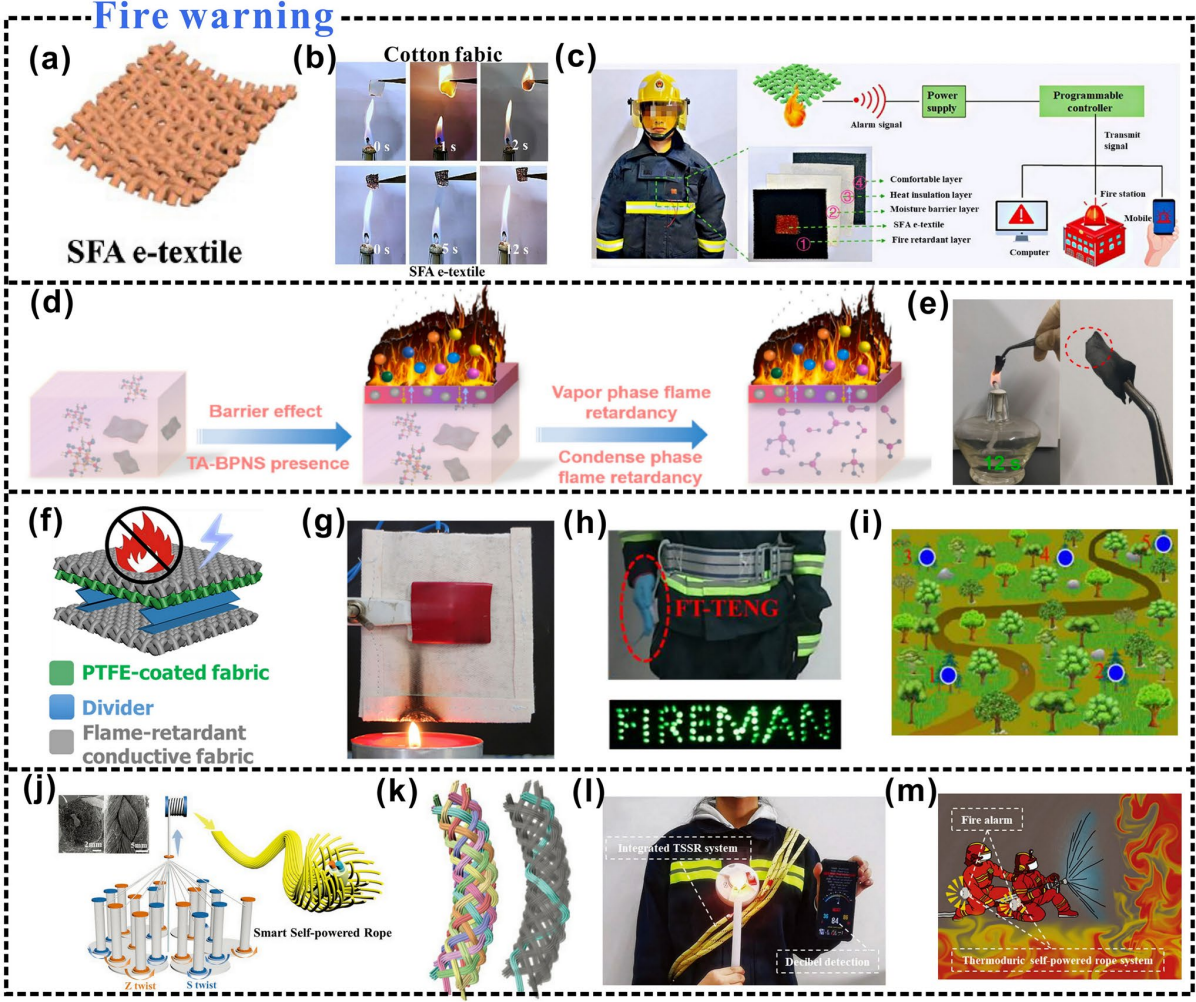
Self-powered wearable sensors for intelligent fire warning. **a** SFA e-textiles. **b** Vertical burning test process of SFA e-textiles under the flame of an alcohol lamp. **c** The working mode of SFA e-textiles as fire alarm materials for fire protective clothing. Reproduced with permission from Ref. [52]. Copyright 2022, American Chemical Society. **d** Illustration of the flame retardancy mechanism of the CNF-BP-PA film. **e** CNF-BP-PA film under open flame burning. Reproduced with permission from Ref. [271]. Copyright 2022, Elsevier. **f** Schematic illustration of the flame-retardant textile-based triboelectric nanogenerator. **g** Combustion photos of FT-TENG. **h** FT-TENG conducts energy harvesting for sending distress signals. **i** Forest map for real-time rescue location positioning. Reproduced with permission from Ref. [63]. Copyright 2020, American Chemical Society. **j** Self-powered tether for smart wearable sensing. **k** Smart self-powered rope made by weaving technology. **l** Emergency rescue in dangerous and extreme environments. **m** Self-powered ropes for fire warning. Copyright 2022, Wiley–VCH

In response to the expansion of portable sensor application scenarios, researchers have conducted detailed studies on the effectiveness of materials over a wide range of temperature variations [269]. Fire safety and combustible prevention are critical in modern society, which has been a global challenge. Frequent fires will cause a large number of casualties and irreparable property losses, which will hurt the global environment. The development of environmentally friendly intelligent fire early warning materials and fire early warning sensors has attracted more and more attention [270]. Wearable sensors are known for their portability and high sensitivity, not only for their material design and production but also for their real-time sensing characteristics. Therefore, wearable fire early warning sensors would be an interesting combination of active and passive fire prevention strategies, and smart fire early warning systems can be prepared by improving the flame retardancy of materials or monitoring the risk of critical fires [264]. He et al. [52], prepared SFA electronic textiles by combining cotton fabric with polytetrafluoroethylene (PTFE) for self-powered fire self-rescue positioning system (Fig. 18a). SFA electronic textiles can resist the burning of an open flame in the vertical test and have good flame retardancy (Fig. 18b). When the SFA e-textiles were exposed to fire, the alarm light was triggered rapidly within 2–3 s, and the current reached above 0.003 A, indicating that the SFA e-textiles exhibited an ultra-sensitive fire alarm response when exposed to fire. SFA electronic textiles can quickly trigger the fire alarm of fire protective clothing by monitoring the temperature rise process (Fig. 18c).

Nanocellulose has an excellent aspect ratio, endowing it with good flexibility, transparency, and elasticity, so it has a good advantage in the preparation of portable wearable sensors [265]. Extreme operating environments pose great challenges to the stable output and sense of triboelectric materials, and cellulosic triboelectric materials have been proven to be able to cope with extreme temperature changes [266]. Combining pure cellulose membrane with biocompatible black phosphorus (BP) and phytic acid (PA), a single-electrode cellulosic TENG (FR-TENG) with flame retardant properties was fabricated with fast response to temperature changes (Fig. 18d) [272]. And the single-electrode cellulosic TENG can preserve the integrity of the material under open flame burning (Fig. 18e). The single-electrode cellulosic TENG has the performance of high-sensitivity temperature response, which expands its application in fire warning.

Textile-based wearable smart sensors have gradually attracted widespread attention, combining the functionality of self-powered sensing with the advantages of breathability and flexibility. Cheng et al. [63], developed a low-cost and environmentally friendly flame-retardant textile-based triboelectric nanogenerator (FT-TENG) by combining flame-retardant conductive cotton fabric, PTFE-coated cotton fabric and separator (Fig. 18f). The textile-based triboelectric nanogenerator has obvious flame retardancy (Fig. 18g), and can be installed on firefighters for energy harvesting to drive LED lights to send distress signals (Fig. 18h). In addition, self-powered sensors can also be used in forest self-rescue and fire alarm systems (Fig. 18i).

When the actual danger comes, it is very important to shorten the rescue time and ensure the safety and health of the human body. Based on the development of wearable technology, it will be an important breakthrough to prepare sensors with practical utility and self-powered characteristics in special extreme environments. At present, smart self-powered ropes have been prepared for smart wearable sensing (Fig. 18j) [273]. The fiber-based zinc battery unit is integrated with the traditional rope unit through weaving technology to make a smart self-powered rope (Fig. 18k). The rope can be used not only for emergency rescue in dangerous and extreme environments (Fig. 18l), but also for fire warning systems (Fig. 18m). The innovation of flame-retardant materials and the development of self-powered wearable sensors have promoted the progress of fire warning sensors and added new vitality to intelligent fire warning.

## Conclusions and Prospects

With the booming development of electronic technology, wireless electronic devices are gradually evolving towards device miniaturization, portability, and multi-functionality. The emergence of self-powered wearable electronics has improved the problems of long charging times and high energy consumption of today's wireless electronic devices. In this article, recent advances in cellulosic triboelectric materials for self-powered wearable electronics are reviewed and highlighted. Cellulosic self-powered wearable electronics solve the poor portability problem of many planar electronics and have the advantages of good flexibility, strong comfort, and multi-functionality. Despite some progress, the response stability of self-powered wearable electronics still deserves attention. Pursuing integrated strategies of novel materials and sensing technologies is an effective way to realize the full potential of self-powered electronics in wearable sensing.

### Optimization of Triboelectric Properties during Preparation

Compared with ordinary modification methods, different modification methods are used in the process of preparing cellulosic triboelectric materials, such as graft modification, and physical doping, which will directly or indirectly affect the cellulosic triboelectric properties. This will affect the sensing effect of self-powered wearable sensors. In the preparation process of top-down, bottom-up and composite materials, different modification methods can be used to endow cellulosic triboelectric materials with more functional properties. For example, improving the triboelectric properties of cellulosic triboelectric materials through physical doping [129], preparation of flame-retardant cellulosic triboelectric materials by vacuum filtration [272], preparation of waterproof layers of cellulosic materials using hydrophobic modification [78]. In addition, exploring the integration of material preparation methods and sensing technology can not only fully improve the triboelectric performance but also fully demonstrate the application potential of cellulosic materials in the wearable field. It also provides interesting insights into the same types of natural polymers (e.g., starch, chitosan, protein, and chitin), stimulating the development potential of self-powered wearable electronics.

### Challenges of Material Preparation Strategies and Environmental Friendliness

Cellulose, as a typical natural polymer macromolecule, has been increasingly used in self-powered sensors in recent years. At present, most cellulosic materials are prepared with highly toxic and refractory chemicals to obtain high-performance materials. It is of great significance to discuss the influence of interfacial properties of materials before molding for the regulation of triboelectric properties. For example, the dispersion of cellulosic materials before molding discussed in the paper affects the triboelectric output. Exploring the influencing factors of triboelectric output can help researchers to choose effective preparation paths, which will help to reduce the use of highly toxic and hard-to-degrade chemicals and reduce the environmental burden. In addition, the modification of cellulose in aqueous medium is a promising direction for the preparation of cellulosic triboelectric materials, such as surface coating under aqueous conditions and grafting under aqueous conditions. The water-based reaction can avoid the use of organic solvents and reduce the harm to the operator and the environment. Different preparation strategies for the aqueous reaction of cellulose can provide an effective solution for the preparation and performance modulation of cellulosic triboelectric materials. Different preparation strategies can provide effective solutions for the preparation of cellulosic triboelectric materials as well as for property modulation.

### Expand the Scope of Application

Cellulose has excellent biocompatibility, and its internal structure is extremely important to perform some specific functions. Common contact electrification types are generally divided into solid–solid contact electrification [274] and solid–liquid contact electrification [271, 275, 276]. Cellulosic self-powered wearable sensors are widely used among solid–solid contact types. As we all know, electronic skin made of cellulosic materials has the advantage of excellent tactility, and can easily cope with the detection of various mechanical strains, finger touches, health status, and airflow signals, etc. With high sensitivity, it is suitable for the fields of human–computer interaction and soft robotics. However, few current studies have linked cellulosic e-skins with self-powered properties. It has to be said that if the electronic skin is endowed with self-powered characteristics, it will greatly stimulate the research vitality of cellulosic self-powered wearable sensors. At present, the application expansion of cellulosic self-powered wearable sensors is rarely seen among solid–liquid contact electrification types. With the development of the artificial intelligence industry, people have put forward higher requirements for the practicability of smart sensors. Therefore, the development of self-powered wearable sensors is also a research topic worth exploring in special environments such as rainy days, foggy weather, and snowy days.

Although challenges remain, cellulosic self-powered wearable electronics are showing strong dynamism in several emerging applications for their good wearability, portability, and sensitivity, further bringing humans and machines closer together. Self-powered wearable electronic products made of environmentally friendly cellulosic materials will undoubtedly burst into greater vitality in the era of the Internet of Things.
